# Paracingulin recruits CAMSAP3 to tight junctions and regulates microtubule and polarized epithelial cell organization

**DOI:** 10.1242/jcs.260745

**Published:** 2023-05-15

**Authors:** Arielle Flinois, Isabelle Méan, Annick Mutero-Maeda, Laurent Guillemot, Sandra Citi

**Affiliations:** Department of Molecular and Cellular Biology, Faculty of Sciences, University of Geneva, 1205 Geneva, Switzerland

**Keywords:** Paracingulin, CAMSAP3, Epithelial cells, Microtubules

## Abstract

Paracingulin (CGNL1) is recruited to tight junctions (TJs) by ZO-1 and to adherens junctions (AJs) by PLEKHA7. PLEKHA7 has been reported to bind to the microtubule minus-end-binding protein CAMSAP3, to tether microtubules to the AJs. Here, we show that knockout (KO) of CGNL1, but not of PLEKHA7, results in the loss of junctional CAMSAP3 and its redistribution into a cytoplasmic pool both in cultured epithelial cells *in vitro* and mouse intestinal epithelium *in vivo*. In agreement, GST pulldown analyses show that CGNL1, but not PLEKHA7, interacts strongly with CAMSAP3, and the interaction is mediated by their respective coiled-coil regions. Ultrastructure expansion microscopy shows that CAMSAP3-capped microtubules are tethered to junctions by the ZO-1-associated pool of CGNL1. The KO of CGNL1 results in disorganized cytoplasmic microtubules and irregular nuclei alignment in mouse intestinal epithelial cells, altered cyst morphogenesis in cultured kidney epithelial cells, and disrupted planar apical microtubules in mammary epithelial cells. Together, these results uncover new functions of CGNL1 in recruiting CAMSAP3 to junctions and regulating microtubule cytoskeleton organization and epithelial cell architecture.

## Teaser

**Summary:** The junctional protein CGNL1 controls the localization of CAMSAP3 at apical epithelial junctions, as well as the organization of microtubules and the polarized architecture of epithelial cells *in vitro* and *in vivo*.

## INTRODUCTION

Epithelial tissues line all body surfaces and cavities, and are formed by polarized cells that interact with each other through specialized cell–cell junctions. The apical junctional complex (AJC) of epithelial cells comprises tight junctions (TJs) and adherens junctions (AJs) and is implicated in cell–cell adhesion, in the generation of a semi-permeable barrier for tissue sheets and in the establishment and maintenance of apico-basal polarity (Meng et al., 2008; Buckley and Turner, 2018).

The actin and microtubule cytoskeletons control cell–cell junction assembly and disassembly, and the overall shape, mechanical properties, architectural organization and function of epithelial cells (Pollard and Cooper, 2009; Vasileva and Citi, 2018; Dogterom and Koenderink, 2019; Tsukita et al., 2023). In turn, junctional proteins organize the cytoskeleton by binding to cytoskeletal proteins and to regulators of Rho family GTPases (Citi et al., 2011; Braga, 2017; Vasileva and Citi, 2018). Microtubules are critical for many cellular processes, such as cell division, intracellular trafficking and morphogenesis (Goodson and Jonasson, 2018). In epithelial cells, most microtubules are non-centrosomal and are aligned along the apico-basal axis of polarization, with microtubule minus-ends anchored apically (Bacallao et al., 1989; Toya and Takeichi, 2016). In specific cell types, such as Eph4 mammary epithelial cells, microtubules are also assembled into a network of mixed polarity under the apical membrane [called the planar apical network (PAN)] (Yano et al., 2017). The spatial organization of the microtubule cytoskeleton allows targeted and polarized delivery of cargos, which is crucial for protein sorting, *de novo* lumen formation, cellular organelle distribution and basal positioning of the nucleus (Müsch, 2004; Toya and Takeichi, 2016; Muroyama and Lechler, 2017). The mechanisms that regulate the switch from a radial to an apico-basal microtubule network in epithelial cells have been in part elucidated. During epithelial polarization radial microtubules are initially nucleated at the centrosome (Bellett et al., 2009; Tanaka et al., 2012) and are subsequently released through the microtubule minus-end-binding protein CAMSAP3 (Dong et al., 2017). Both CAMSAP2 and CAMSAP3 bind to growing microtubule minus-ends, stabilize non-centrosomal microtubules and are crucial for anchoring of microtubule minus-ends to the apical submembrane region (Tanaka et al., 2012; Noordstra et al., 2016; Toya and Takeichi, 2016). In addition to the apical anchoring of microtubules, CAMSAP3 has been reported to tether microtubules to AJ of cultured intestinal Caco2 cells, via its interaction with the cytoplasmic junctional protein PLEKHA7 (Meng et al., 2008). However, the role of PLEKHA7 in the organization of microtubules and polarized cell architecture in different types of cultured cells and *in vivo* is not clear.

Previously, we independently identified PLEKHA7 as an interactor of paracingulin (CGNL1) (Pulimeno et al., 2010, 2011). CGNL1, also known as junction-associated coiled-coil protein (JACOP) is a protein localized in the cytoplasmic region of both TJs and AJs (Ohnishi et al., 2004; Guillemot and Citi, 2006), where it is recruited by ZO-1 (also known as TJP1) and PLEKHA7, respectively (Pulimeno et al., 2011). CGNL1 is also a paralog of cingulin (CGN), a TJ protein that binds to microtubules to organize the PAN of microtubules in Eph4 cells and regulate apical lumen formation in MDCK cells (Yano et al., 2013; Mangan et al., 2016). Interestingly, CGNL1 interacts with microtubules *in vitro* (Vasileva and Citi, 2018); however, its role in the regulation of microtubule organization is not known. Here, we show that CGNL1, but not PLEKHA7, recruits CAMSAP3 to TJs, and that CGNL1 is involved in the organization of the non-centrosomal microtubule network and polarized epithelial cell architecture in cultured cells and *in vivo*.

## RESULTS

### CGNL1 is required for the junctional localization of CAMSAP3 in kidney collecting duct and mammary epithelial cells

**Fig. 1.**
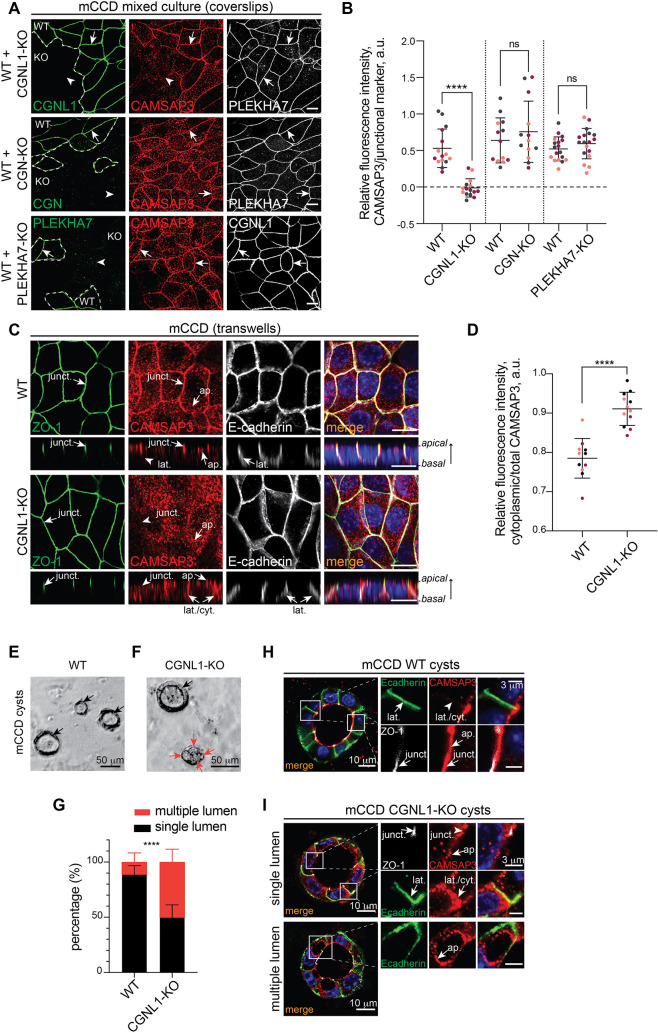
**CGNL1 recruits CAMSAP3 to apical junctions of epithelial mCCD cells.** (A) Immunofluorescence microscopy analysis of the localization of CAMSAP3 (red) in mixed cultures of mCCD WT cells and cells KO for CGN, CGNL1 or PLEKHA7 cells, using PLEKHA7 or CGNL1 as junctional markers (white). PLEKHA7, CGN or CGNL1 (green) are used to distinguish WT from KO cells (white dashed line separating WT from KO). Arrows and arrowheads indicate detectable and decreased/undetectable junctional labeling, respectively. Scale bars: 10 µm. (B) Quantification of junctional labeling intensity for CAMSAP3, normalized to either PLEKHA7 [for WT+CGNL1-KO (*n*=15) and WT+CGN-KO (*n*=13) cells], or to CGNL1 (WT+PLEKHA7-KO (*n*=18) cells). Bars represent mean±s.d. for three biological replicates. ****P<0.0001; ns, not significant (unpaired two-tailed *t*-test). (C) Immunofluorescence microscopy analysis of the localization of CAMSAP3 (red) in either mCCD WT or CGNL1-KO cells grown on Transwell inserts. ZO-1 (green) was used as a TJ marker, and E-cadherin (white) as an AJ and lateral marker. *Z*-sections were taken at the horizontal middle positions and are shown below *XY* images. Junctional (junct.), apical (ap.), lateral (lat.) and cytoplasmic (cyt.) localizations are indicated by arrows. Scale bars: 10 µm. (D) Quantification of labeling intensity of CAMSAP3 in the cytoplasm, ratioed to the total CAMSAP3 signal in WT (*n*=11) or CGNL1-KO (*n*=12) cells. Bars represent mean±s.d. for three biological replicates. ****P<0.0001 (unpaired two-tailed *t*-test). (E,F) Phase-contrast images of cysts for mCCD WT (E) and CGNL1-KO (F) cells. Black arrows indicate a unique lumen and red arrows indicate multiple lumens within the same cyst. Scale bars: 50 µm. (G) Quantification of 3D cyst formation of mCCD WT or CGNL1-KO cells as shown in E,F. Percentage of cysts with a single lumen (black) and multiple lumens (red) are plotted for each cell line. Bars represent mean±s.d. for three biological replicates. WT, *n*=290 cysts; CGNL1-KO, *n*=223 cysts. ****P<0.0001 (two-way ANOVA with post hoc Sidak's test). (H,I) Immunofluorescence microscopy analysis of the localization of CAMSAP3 (red) in either mCCD WT (H) or CGNL1-KO (I) cysts. E-cadherin (green) is used as an AJ and lateral marker, ZO-1 (white) as a TJ marker. Arrows are as for C. Magnified images on the right show details of apical and lateral area outlined by square boxes. Scale bars: 10 µm (main images); 3 µm (magnifications). a.u., arbitrary units.

Given that CGNL1 interacts with PLEKHA7 (Pulimeno et al., 2011) and microtubules (Vasileva and Citi, 2018), we wondered whether CGNL1 could modulate the junctional recruitment of CAMSAP3 by PLEKHA7 and orchestrate microtubule organization. To this purpose, we first analyzed mixed cultures of wild-type (WT) cells and CGNL1-knockout (KO) epithelial cells (either mCCD, derived from the kidney collecting duct, or Eph4, derived from the mammary gland) (Vasileva et al., 2022) by immunofluorescence with previously validated (Meng et al., 2008; Tanaka et al., 2012) antibodies against endogenous CAMSAP3 (Fig. 1; Fig. S1). For comparison, we also analyzed CGN KO cells (mCCD and Eph4) (Vasileva et al., 2022) and PLEKHA7 KO cells (mCCD) (Shah et al., 2018). In confluent WT cells, CAMSAP3 labeling was clearly detectable at junctions, colocalizing with PLEKHA7 (white arrows in Fig. 1A and Fig. S1A). In contrast, in CGNL1-KO cells, CAMSAP3 labeling at junctions was either significantly decreased or undetectable (white arrowheads in Fig. 1A and in Fig. S1A, top panel; quantification in Fig. 1B) in both cell types. Junctional CAMSAP3 was rescued by re-expression of CGNL1 (Fig. S1B, top panel) but not of CFP (Fig. S1B, bottom panel). In contrast, in CGN-KO mCCD and Eph4 cells, the junctional localization of CAMSAP3 was similar to that in the WT cells (arrows in Fig. 1A, middle panel, and Fig. S1A, bottom panel; quantification in Fig. 1B). Surprisingly, although the depletion of PLEKHA7 was previously reported to result in decreased CAMSAP3 localization at junctions of Caco2 cells (Meng et al., 2008), the junctional localization of CAMSAP3 was not affected by KO of PLEKHA7 in mCCD cells (arrow in Fig. 1A, bottom panel; quantification in Fig. 1B). Also, despite the previously established role of PLEKHA7 in the recruitment of CGNL1 in MDCK cells (Pulimeno et al., 2011), CGNL1 junctional labeling was not decreased in PLEKHA7-KO mCCD cells (arrows in Fig. 1A, bottom panel), suggesting that, in these cells, CGNL1 is mostly localized at TJs.

To analyze in further detail the role of CGNL1 in the localization of CAMSAP3, mCCD cells were grown on Transwell filters, to obtain maximal cell polarization. Analysis of *z*-sections showed that, in WT cells, CAMSAP3 was detectable not only at junctions (‘junct.’ arrows in Fig. 1C, WT) but also in the cytoplasm near the apical cortex (‘ap.’ arrows in Fig. 1C, WT), in agreement with similar observations on intestinal cells (Noordstra et al., 2016; Toya and Takeichi, 2016). The KO of CGNL1 resulted in the loss of CAMSAP3 junctional labeling (‘junct.’ arrowhead in Fig. 1C, CGNL1-KO) and in the increased CAMSAP3 cytoplasmic labeling along the lateral borders, when compared to WT cells (‘lat./cyt.’ arrows in Fig. 1C, lateral view of CGNL1-KO; quantification in Fig. 1D). Instead, apical CAMSAP3 labeling was similar in WT and CGNL1-KO cells (‘ap.’ arrows Fig. 1C).

Finally, we analyzed the role of CGNL1 in regulating the localization of CAMSAP3 in cells grown in Matrigel, which form cysts. WT mCCD cysts typically contained one single lumen (black arrows in Fig. 1E; quantification in Fig. 1G). Instead, only ∼50% of CGNL1-KO cysts had a single lumen, the remaining 50% contained two or more lumens (multiple red arrows in Fig. 1F; quantification in Fig. 1G). In WT cysts, CAMSAP3 was clearly detectable at junctional and apical localizations, this latter bordering the lumen (‘junct.’ and ‘ap.’ arrows, respectively, in Fig. 1H), whereas it was undetectable near the lateral borders, identified by the E-cadherin labeling (‘lat.’ arrowhead, Fig. 1H). In CGNL1-KO cysts, in contrast, junctional CAMSAP3 was decreased or undetectable (‘junct.’ arrowhead in Fig. 1I), whereas increased CAMSAP3 labeling was detected in the cytoplasm, especially near the lateral border of polarized cells, proximal to but not overlapping with E-cadherin labeling (‘lat./cyt.’ arrows in Fig. 1I). Apical localization of CAMSAP3 was still detected in CGNL1-KO cysts, bordering single and ectopic lumen (‘ap.’ arrows in Fig. 1I). To determine whether decreased junctional localization of CAMSAP3 was due to degradation, we carried out immunoblot analysis of CAMSAP3 levels. In both mCCD and Eph4 cell lysates, levels of expression of CAMSAP3 were similar in WT and CGNL1-KO cells (Fig. S2A,B; quantification in Fig. S2C–E).

Collectively, these results show that the KO of CGNL1, but not of either PLEKHA7 or CGN, results in the loss of the junctional localization of CAMSAP3, and the redistribution of junctional CAMSAP3 to a cytoplasmic and lateral localization. In contrast, the KO of CGNL1 does not affect the apical localization of CAMSAP3.

### The ZO-1-associated pool of CGNL1 recruits CAMSAP3 to tight junctions

**Fig. 2.**
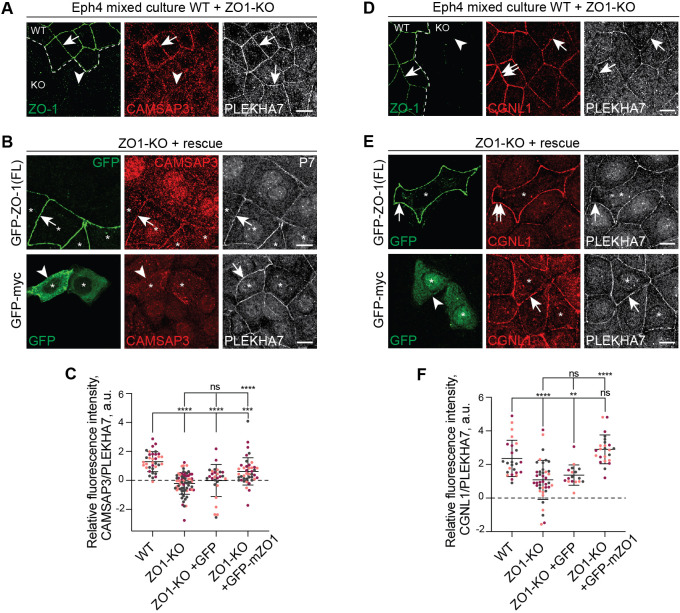
**CAMSAP3 is recruited to junctions by the ZO-1-associated pool of CGNL1.** (A,B) Immunofluorescence microscopy analysis of CAMSAP3 (red) in mixed cultures of WT and ZO1-KO Eph4 cells (A) and in ZO1-KO Eph4 cells rescued with GFP-tagged ZO-1 (full-length, top) or GFP alone (negative control, bottom) (B). PLEKHA7 (white) is used as a junctional marker, and ZO-1 (green) to distinguish between WT and KO cells (white dashed line). Arrows indicate junctional labeling; arrowheads indicate reduced or undetectable junctional labeling; double arrows indicate increased junctonal labeling. Asterisks indicate transfected cells. The nuclear labeling in Fig. 2B is likely due to non-specific cross-reaction of primary or secondary antibodies. Scale bars: 10 µm. (D,E) Immunofluorescence microscopy analysis of the labeling intensity of CGNL1 (red) in mixed cultures of WT and ZO1-KO Eph4 cells (D) and in ZO1-KO Eph4 cells rescued with GFP-tagged ZO-1 (full-length, top) or GFP alone (negative control, bottom) (E). PLEKHA7 (white) is used as a junctional marker, and ZO-1 (green) to distinguish between WT and KO cells (white dashed line). Double arrows indicate increased junctional labeling. Scale bars: 10 µm. (C,F) Quantification of junctional labeling intensity for CAMSAP3 (C) or CGNL1 (F), normalized to PLEKHA7 in WT [*n*=36 (C) or 29 (F)], ZO1-KO [*n*=74 (C) or 45 (F)] or ZO1-KO rescued with GFP [*n*=30 (C) or 18 (F)] or GFP-tagged ZO-1 [*n*=44 (C) or 27 (F)]. Bars represent mean±s.d. for three biological replicates. **P<0.01; ***P<0.001; ****P<0.0001; ns, not significant (one-way ANOVA with post hoc Dunnett's test). a.u., arbitrary units.

PLEKHA7 and ZO-1 recruit CGNL1 to AJs and TJs, respectively (Pulimeno et al., 2011). Given that the KO of PLEKHA7 in mCCD cells did not affect the junctional localization of either CAMSAP3 or CGNL1 (Fig. 1A), we hypothesized that in these cells CGNL1 is mostly associated with TJs, through interaction with ZO-1. To test this hypothesis, we asked whether the KO of ZO-1 affected junctional CAMSAP3 labeling. Immunofluorescence microscopy analysis showed that CAMSAP3 labeling was decreased at junctions of ZO-1-KO Eph4 cells, as compared to that in WT cells, although a faint peripheral labeling was still detectable (arrowhead in Fig. 2A; quantification in Fig. 2C). Junctional CAMSAP3 was rescued by re-expression of full-length ZO-1, but not GFP alone (arrows and arrowhead in Fig. 2B; quantification in Fig. 2C). In addition, junctions of ZO-1-KO cells showed decreased CGNL1 labeling at junctions (single arrow in Fig. 2D), which was rescued by expression of ZO-1 but not by GFP (double and single arrows, respectively in Fig. 2E; quantification in Fig. 2F).

These results suggest that the TJ-associated pool of CGNL1, which is recruited by ZO-1, is primarily involved in the junctional recruitment of CAMSAP3.

### Microtubule-CAMSAP3 tethering to tight junctions and apical microtubule organization are disrupted in CGNL1-KO cells

Previous studies have reported that CAMSAP3 mediates microtubule tethering to AJs (Meng et al., 2008) and nucleates cytoplasmic microtubules to influence their pattern of assembly (Tanaka et al., 2012). Given that CGNL1 recruits CAMSAP3 to TJs (Figs 1, 2), we hypothesized that KO of CGNL1 could affect microtubule tethering to junctions and alter microtubule organization. To test this hypothesis, we used ultrastructure expansion microscopy (U-ExM) (Gambarotto et al., 2019) and STED microscopy, which allow super-resolution imaging of the microtubule network.

**Fig. 3.**
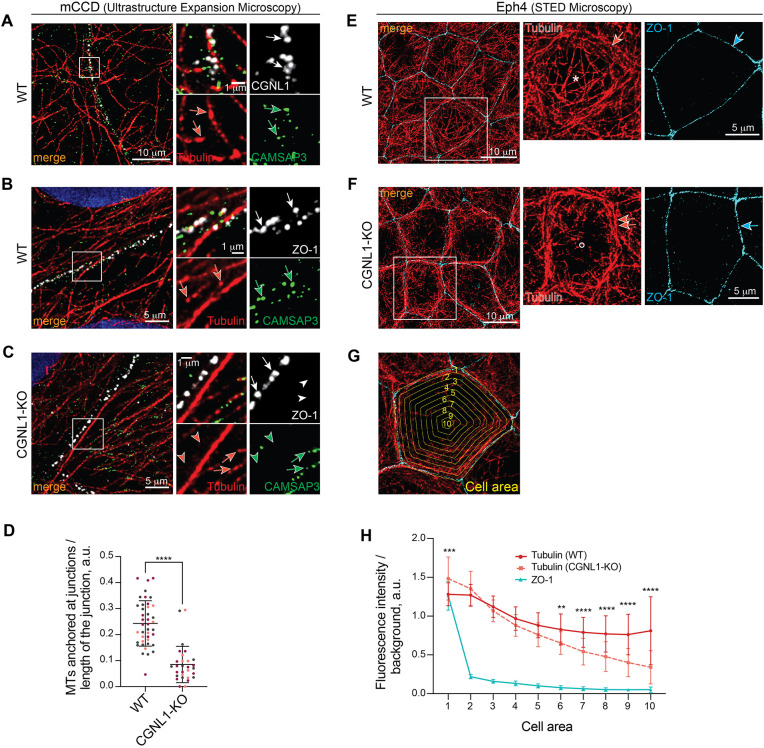
**CGNL1 anchors microtubule minus-ends at junctions and is required for the proper microtubule apical organization in Eph4 cells.** (A) Immunofluorescence microscopy analysis after expansion (U-ExM) of the microtubule network (tubulin, red), CAMSAP3 (green) and CGNL1 (white) in mCCD WT cells (close-up next to a zonular junction). The magnified view on the right shows details of area outlined by a square box. Arrows (CGNL1, white; CAMSAP3, green; tubulin, red) show close proximity between labeling. Scale bars: 10 and 1 µm in low and high magnification, respectively. (B,C) Immunofluorescence microscopy analysis after expansion (U-ExM) of the microtubule network (tubulin, red) and CAMSAP3 (green) in mCCD WT (B) or CGNL1-KO (C) cells. ZO-1 (white) is used as a tight junction marker. Insets on the right show high magnification details of the area outlined by a square box. Arrows and arrowheads (ZO-1, white; CAMSAP3, green; tubulin, red) indicate close or absence of proximity, respectively. Scale bars: 10 µm (main images); 1 µm (magnifications). (D) Quantification of the number of CAMSAP3-capped microtubule-ends within 0.2 µm of the junction (1 µm expanded) normalized to the length of the junction in WT (*n*=42 junctional segments) or CGNL1-KO mCCD cells (*n*=29). Bars represent mean±s.d. for three biological replicates. ****P<0.0001 (unpaired two-tailed *t*-test). (E,F) Immunofluorescence microscopy analysis (STED) of the planar apical network of microtubules in WT (E) and CGNL1-KO (F) Eph4 cells. Microtubules are labeled with tubulin (red), ZO-1 (cyan) is used as a junctional marker. The magnified views on the right show details of areas outlined in square boxes. Asterisks and circles indicate homogenous apical microtubule network and absence of microtubules, respectively. Arrows and double arrows indicate normal and increased association of apical microtubules with junctions, respectively. Scale bars: 10 µm (main images); 5 µm (magnifications). (G) Representation of the segmentation of the cell for quantification. Areas (yellow) are labeled from 1 to 10 starting from the periphery to the center of the cell. (H) Quantification of labeling intensity for tubulin in the respective cell areas (1 to 10, as shown in the representation in G) for WT (red, *n*=22 cells) and CGNL1-KO (light red, *n*=24 cells) cells. ZO-1 (cyan) is plotted to mark junction position. Bars represent mean±s.d. for three biological replicates. **P<0.01, ***P<0.001, ****P<0.0001 (two-way ANOVA with post hoc Sidak's test). a.u., arbitrary units.

WT mCCD cells were labeled with antibodies against tubulin, CAMSAP3 and CGNL1 to determine the spatial proximity of CAMSAP3-capped microtubules to the CGNL1–ZO-1 complex at TJ. U-ExM showed that in the junctional regions of WT cells, CAMSAP3 signal (green arrows in magnified view, Fig. 3A) was mostly associated with microtubule ends (tubulin, red arrows in magnified view, Fig. 3A) and was in close proximity of CGNL1 labeling (white arrows in magnified view, Fig. 3A). Next, to compare the localization of microtubules and CAMSAP3 in WT versus CGNL1-KO cells, we examined the relative positions of CAMSAP3 and microtubule signals with respect to ZO-1 (Fig. 3B,C). In WT cells, CAMSAP3 and microtubule labeling was detected peri-junctionally, in the same spatial region as ZO-1 labeling (white, red and green arrows in magnified views, Fig. 3B). In contrast, in CGNL1-KO cells, CAMSAP3 and tubulin labeling were colocalized (green and red arrows, magnified view in Fig. 3C), but were spatially separate from junctional ZO-1 labeling (white arrowheads in magnified view, Fig. 3C). Quantitative analysis of the number of CAMSAP3-capped microtubule ends localized in the peri-junctional area showed a significant decrease in CGNL1-KO cells (Fig. 3D).

To further examine the role of CGNL1 in the organization of microtubules, we undertook STED microscopy of the PAN, which is typical of Eph4 cells (Yano et al., 2013). In WT cells, microtubule labeling was distributed in a homogeneous pattern throughout the apical region and near ZO-1-labeled TJs (asterisks and arrows in magnified views, respectively, in Fig. 3E). Strikingly, peri-junctional tubulin labeling was increased in CGNL1-KO cells (double arrows in magnified view in Fig. 3F), correlating with a decrease in labeling in the central cytoplasm (white circle in magnified view in Fig. 3F) suggesting a redistribution of apical microtubules from the center to the periphery. To quantify these observations, we segmented cells into ten concentrical zones from the periphery to the center, and analyzed fluorescence intensity for tubulin and ZO-1 in each of these areas (Fig. 3G; quantification in Fig. 3H). ZO-1 labeling was detected exclusively in the junctional area, and tubulin labeling was significantly increased in the outer peri-junctional areas of the cell in CGNL1-KO cells (area 1, Fig. 3H). In contrast, the intensity of tubulin was significantly decreased in the central areas of the cell in CGNL1-KO cells (areas 6–10, Fig. 3H).

Together, these results indicate that CGNL1 binds to CAMSAP3 and ZO-1 to tether microtubule minus ends to TJs and promotes the homogenous distribution of the PAN of microtubules in Eph4 cells.

### The KO of CGNL1 but not of PLEKHA7 alters the localization of CAMSAP3 in mouse epithelia *in vivo*

To establish the physiological relevance of our observations, we generated mice KO for either CGNL1 or PLEKHA7, and examined CAMSAP3 localization, epithelial cell architecture and microtubule organization in WT and KO epithelial tissues. To generate CGNL1-KO mice, the CGNL1 locus was targeted by homologous recombination in embryonic stem cells (ESCs), to insert LoxP sites flanking exons 1–2 of CGNL1 (Fig. S3A, targeted allele). The mutated KO allele (–) was generated by Cre-mediated recombination (Fig. S3A, mutated allele). Alleles with targeted and mutated sequences were identified by Southern blotting and PCR (Fig. S3B,C). Immunoblot and immunofluorescence microscopy analysis of homozygous mutant (−/−) mice showed loss of CGNL1 labeling in tubular cells of kidney tissue (Fig. S3D, and arrow and arrowhead in magnified view ‘T’ in Fig. S3E). PLEKHA7-KO mice were generated by CRISPR-Cas9 genome editing, resulting in a 2-bp insertion in exon 17 (Fig. S4A,B). Immunoblot and immunofluorescence microscopy analysis of homozygous mutant (−/−) mice showed loss of PLEKHA7 protein in kidney lysates (Fig. S4C), and loss of PLEKHA7 labeling in kidney epithelial cells (arrow and arrowhead in Fig. S4D).

**Fig. 4.**
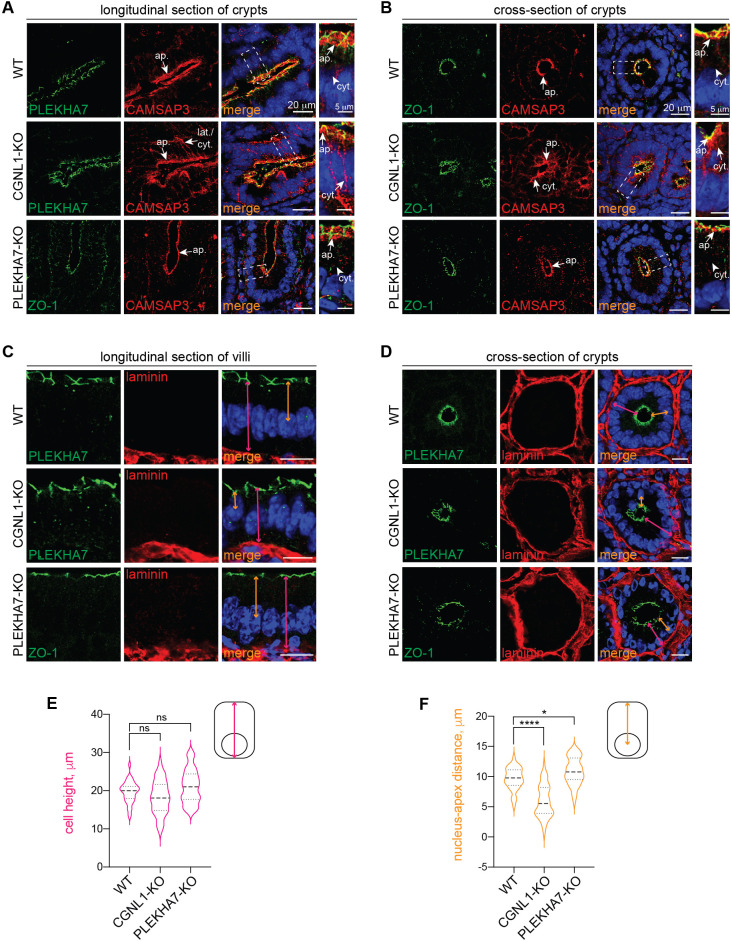
**The KO of CGNL1 alters distribution of CAMSAP3 and polarized epithelial architecture in intestinal epithelial cells *in vivo*.** (A,B) Immunofluorescence microscopy analysis of the localization of CAMSAP3 (red) from either longitudinal (A) or cross-sections (B) of crypts in intestine tissue from WT (top panels) or CGNL1-KO (middle panels) or PLEKHA7-KO (bottom panels) mice. PLEKHA7 or ZO-1 (green) are used as junctional markers. Magnified images on the right show details of areas outlined in boxes. Junctional (junct.), apical (ap.), lateral (lat.) and cytoplasmic (cyt.) localizations are indicated by arrows. Scale bars: 20 µm (main images); 5 µm (magnifications). (C,D) Immunofluorescence microscopy analysis of the basal lamina (laminin, red) of either longitudinal sections of villi (C) or cross-section of crypts (D) in intestine tissue sections from WT mice (top panels) or KO mice for either CGNL1 (middle panels) or PLEKHA7 (bottom panels). PLEKHA7 or ZO-1 (green) are used as junctional markers. Double-headed pink arrows indicate cell height and double-headed orange arrows indicate nucleus–apex distance. Scale bars: 10 µm. (E,F) Quantification of the height [E, pink, *n*=35 (WT), 40 (CGNL1-KO) or 59 cells (PLEKHA7-KO)] and distance between the nucleus and the apex [F, orange, *n*=36 (WT), 53 (CGNL1-KO) or 48 cells (PLEKHA7-KO)] in intestinal cells of WT, CGNL1-KO and PLEKHA7-KO cells. Presented as a violin plot, with median and quartiles, for three biological replicates. *P<0.05; ****P<0.0001; ns, not significant (unpaired two-tailed *t*-test).

Frozen sections of intestine from WT and CGNL1-KO mice were analyzed by immunofluorescence microscopy with antibodies against either PLEKHA7 or ZO-1, as markers for the AJC, and CAMSAP3. In intestinal epithelial cells of WT crypts sectioned either longitudinally (Fig. 4A) or by cross-section (Fig. 4B) CAMSAP3 labeling was detected almost exclusively in the apical region of the cells (‘ap.’ arrows in magnified views, Fig. 4A,B, WT), in agreement with previous reports (Toya et al., 2016). The apical CAMSAP3 labeling was partially colocalized with labeling for PLEKHA7 (Fig. 4A) and ZO-1 (Fig. 4B), suggesting that CAMSAP3 is also localized at apical junctions *in vivo*. In contrast, in CGNL1-KO intestinal cells, CAMSAP3 labeling was detected not only apically, but also laterally and in the cytoplasm (‘cyt.’ arrow in magnified views, Fig. 4A,B, CGNL1-KO), suggesting a redistribution of a fraction of CAMSAP3 to the cytoplasm. In PLEKHA7-KO mice, CAMSAP3 localization was exclusively in the apical region, similar to what is seen for WT tissues (‘ap’ arrow in Fig. 4A,B, PLEKHA7-KO).

Because KO of CAMSAP3 results in altered epithelial architecture (Toya et al., 2016), we examined cell morphology in intestinal and kidney epithelial tissues. Longitudinal and cross-sections of intestinal villi and crypts from WT, CGNL1-KO and PLEKHA7-KO mice were immunofluorescently labeled with antibodies against a junctional marker (either PLEKHA7 or ZO-1) and a basal lamina marker, laminin (Fig. 4C and Fig. 4D). To assess polarized architecture, we measured (1) epithelial cell height, defined as the distance between junctional PLEKHA7 and laminin labeling (pink double-headed arrow in scheme of Fig. 4E), and (2) nucleus–apex distance, defined as the distance between the geometrical center of DAPI-labeled nuclei and apical PLEKHA7 labeling (orange double-headed arrow in scheme of Fig. 4F). In WT and PLEKHA7-KO tissues, nuclei were aligned along the base of the cells, proximal to the basal lamina, with a nucleus–apex distance of ∼10 µm (orange double-headed arrows in Fig. 4C,D, WT and PLEKHA7-KO; quantification in Fig. 4F). In contrast, in CGNL1-KO tissues, nuclei were not aligned, but showed a random distribution, with some nuclei ectopically shifted to the apical region of the cell, and an average nucleus–apex distance of ∼5 µm (orange double-headed arrows in Fig. 4C,D, CGNL1-KO; quantification in Fig. 4F). The average height of WT, PLEKHA7-KO and CGNL1-KO intestinal epithelial cells was similar (∼19.65 µm in WT, ∼21.13 µm in PLEKHA7-KO and ∼18.27 µm in CGNL1-KO cells, pink double-headed arrows in Fig. 4C,D); however, CGNL1-KO cells had a wider range of heights, between 10 and 28 µm (quantification in Fig. 4E). Next, we analyzed CAMSAP3 localization in the kidney cortex. In WT kidney tubules, CAMSAP3 labeling was partially colocalized with PLEKHA7 in the apical region of polarized epithelial cells (arrows in Fig. S5A, WT). In CGNL1-KO kidney tissue, CAMSAP3 colocalization with PLEKHA7 was significantly decreased (arrowheads in Fig. S5A, CGNL1-KO; quantification in Fig. S5B). Instead, colocalization of CAMSAP3 with ZO-1 in PLEKHA7-KO kidney tissues was not altered compared to WT mice (arrows in Fig. S5A, PLEKHA7-KO, quantification in Fig. S5B). CAMSAP3 labeling was also prominent along ciliary-like structures in WT and both CGNL1- and PLEKHA7-KO tissues (red arrows in Fig. S5A, magnified views), suggesting a localization at the primary cilium of tubular epithelial cells. To test this hypothesis, we labeled kidney sections with antibodies against poly-glutamylated (polyE) tubulin, which is enriched at basal bodies of cilia (Wloga et al., 2017). Immunofluorescence microscopy showed polyE labeling at the base of elongated structures in the apical region of kidney epithelial cells (red and green arrows in magnified views, Fig. S5C), corresponding to the cilium.

**Fig. 5.**
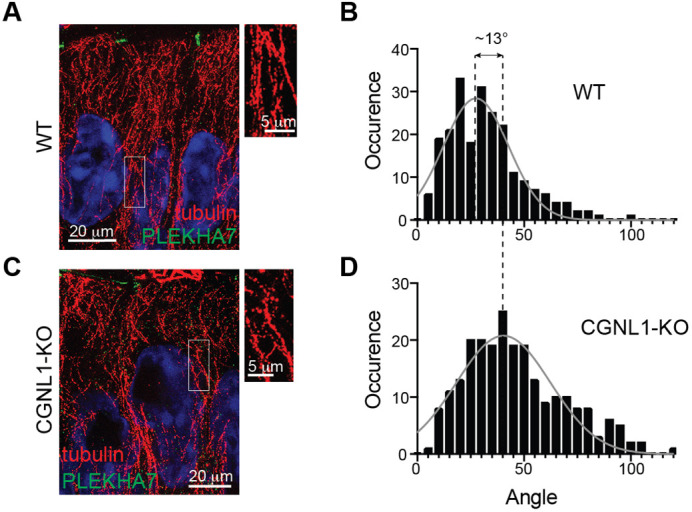
**Organization of the microtubule network in intestinal cells *in vivo*.** (A,C) Immunofluorescence microscopy analysis of microtubules (tubulin, red) in expanded intestine tissue sections from mice either WT (A) or KO for CGNL1 (C). PLEKHA7 (green) is used as a junctional marker, and nuclei are stained in blue. Magnified images on the right show details of areas outlined in boxes. Scale bars: 20 µm (main images); 5 µm (magnifications). (B,D) Quantification of microtubule directionality by analyzing the angle at which microtubules cross an arbitrary line drawn along the apico-basal axis of WT (B, *n*=222 crossing points) or CGNL1-KO (D, *n*=232) cells. The occurrence for each angle is plotted and fitted to a Gaussian curve. Mean for two biological replicates.

Finally, we analyzed the distribution of microtubules in intestinal epithelial cells using U-ExM (Mercey et al., 2022), and measured the tilt angle of individual microtubules with respect to the basal–apical vertical axis (Toya et al., 2016). Tubulin labeling was distributed along the basal–apical axis in WT cells (Fig. 5A), with a mean crossing angle of 27.44° (Fig. 5B), whereas in CGNL1-KO cells microtubules were more randomly oriented (Fig. 5C) with a mean crossing angle of 40.35° (Fig. 5D).

Collectively, our results show that CGNL1, and not PLEKHA7, is required for the junctional localization of CAMSAP3 and for maintaining the polarized architecture of epithelial cells and vertical microtubule alignment *in vivo*, but CGNL1 is not required for the localization of CAMSAP3 at the apical cortex and in cilia.

### CGNL1 and CAMSAP3 interact through their respective coiled-coil domains

**Fig. 6.**
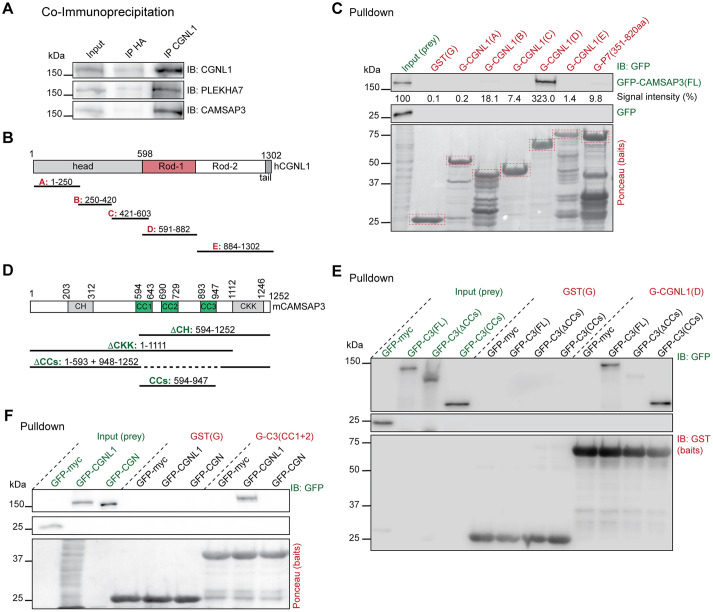
**The coiled-coil Rod-1 region of CGNL1 interacts with the coiled-coil domains of CAMSAP3 *in vitro*.** (A) Immunoblot (IB) analysis, using antibodies against either CGNL1, CAMSAP3 or PLEKHA7, of lysates of mCCD cells (input, 10%) and immunoprecipitates (IP) obtained using either anti-HA (negative control) or anti-CGNL1 antibodies. (B) Simplified scheme of structural domains (head, rod and tail) of human CGNL1 and GST fusion constructs (A to E), indicating amino acid residue boundaries. (C) IB analysis, using anti-GFP antibodies, of GST pulldowns, using the GST–CGNL1 fragments described in B and GST-PLEKHA7(P7) (351-820) as baits (red), and either full-length GFP-tagged CAMSAP3 or GFP (negative control) as preys (green). Baits are shown in a Ponceau-stained image below the IB. Numbers indicate the migration of pre-stained molecular size markers. Signal intensity of GFP–CAMSAP3(FL) (prey) bound to GST-tagged CGNL1 and PLEKHA7 fragments (baits), normalized to input, are displayed below the GFP–CAMSAP3(FL) blot (*n*=3 biological replicates). (D) Scheme of CAMSAP3 structural domains, showing amino acid boundaries: calponin homology (CH), coiled-coil (CC, green), calmodulin-regulated spectrin-associated (CKK). Below, schemes of GFP-tagged CAMSAP3 constructs used for pulldown experiments (in E and Fig. S6B). (E) IB analysis, using anti-GFP antibodies, of GST pulldowns using GST or the CAMSAP3-binding region of CGNL1 (fragment D) as baits, and GFP-tagged preys {full-length CAMSAP3 [GFP-C3(FL)] or CAMSAP3 lacking the three coiled-coil domains [GFP-C3(ΔCCs)], or only the region encoding the three coiled-coil domains [residues 594–947, GFP-C3(CCs)], or only GFP (negative control)}. Baits are shown also by IB analysis with anti-GST antibodies. (F) IB analysis, using anti-GFP antibodies, of GST pulldowns using GST or GST fused to the region comprising the first two coiled-coil domains (residues 600–729) of CAMSAP3 as baits, and GFP (negative control), GFP-tagged full-length CGN (GFP-CGN) or GFP-tagged full-length CGNL1 (GFP-CGNL1) as preys. Baits are shown in Ponceau-stained images below the IB. Blots shown in this figure are representative of three repeats.

To establish the biochemical basis for the regulation of CAMSAP3 by CGNL1, we examined whether CGNL1 and CAMSAP3 interact together. Immunoprecipitation of mCCD lysates with antibodies against CGNL1 showed that both PLEKHA7 and CAMSAP3 form a complex with CGNL1 (Fig. 6A). In addition, a proximity ligation assay (PLA) in mCCD cells showed that CGNL1 and CAMSAP3 are closely colocalized along ZO-1-labeled junctions in WT, PLEKHA7-KO and CGN-KO cells (arrows in Fig. S6A, three top panels). This observation confirmed that neither PLEKHA7 nor CGN are required for the localization of CAMSAP3 at junctions and for CAMSAP3 interaction with CGNL1. The CGNL1–CAMSAP3 PLA signal was undetectable in CGNL1-KO cells (negative control, arrowhead in Fig. S6A, bottom panel).

Next, to test whether CGNL1 interacts with CAMSAP3 *in vitro* and to identify the interacting regions, we first generated GST fusion protein baits encoding fragments of CGNL1 (schemes in Fig. 6B) and examined their interaction with a full-length CAMSAP3 prey. CGNL1 contains a globular head domain, a coiled-coil rod domain comprising N-terminal (Rod-1) and C-terminal (Rod-2) segments, and a small globular tail (Fig. 6B). GST pulldowns showed that the ‘D’ fragment of the coiled coil domain of CGNL1, corresponding to the Rod-1 region (Fig. 6B, residues 591–882) interacted strongly with the full-length, GFP-tagged CAMSAP3 prey, whereas none of the other fragments interacted significantly with CAMSAP3 (Fig. 6C, quantification of signal intensity below blot). To compare the binding of CAMSAP3 to CGNL1 versus PLEKHA7, we used a fragment of PLEKHA7 that was previously shown to bind to CAMSAP3 as a bait [Fig. 6C, GST-P7(351-820)] (Meng et al., 2008). Immunoblot analysis showed that PLEKHA7 interacts very weakly with CAMSAP3, compared to CGNL1 (Fig. 6C, quantification of signal intensity below blot).

To map the region of CAMSAP3 that interacts with CGNL1, the Rod-1 ‘D’ CGNL1 fragment was used as a bait and truncated or mutated GFP-tagged constructs of CAMSAP3 were used as preys (scheme of preys in Fig. 6D). CAMSAP3 contains an N-terminal calponin homology (CH) domain, three central coiled-coil (CC) regions and a C-terminal calmodulin-regulated spectrin-associated (CKK) domain, this latter being responsible for the interaction with microtubules (Fig. 6D) (Toya et al., 2016). Immunoblot analysis showed that deletion of the coiled-coil domains (ΔCCs) of CAMSAP3, resulted in strongly decreased interaction with CGNL1 (Fig. 6E; Fig. S6B). Moreover, a CAMSAP3 prey comprising only the three coiled-coil regions of CAMSAP3 (CCs, residues 594–947, Fig. 6D) interacted strongly with the Rod-1 CGNL1 bait (Fig. 6E). In addition, a GST-tagged bait comprising only the first two coiled-coil domains (CC1+2) interacted strongly with full-length CGNL1, used as a prey, but not with CGN (Fig. 6F).

Together, these experiments show that CGNL1, but neither CGN nor PLEKHA7, interact with CAMSAP3, and that this interaction depends on the coiled-coil Rod-1 domain of CGNL1 and the coiled-coil domains of CAMSAP3.

### The Rod-1 domain of CGNL1 and a 5-amino-acid stretch in the coiled-coil-1 region of CAMSAP3 are required for CAMSAP3 junctional recruitment

**Fig. 7.**
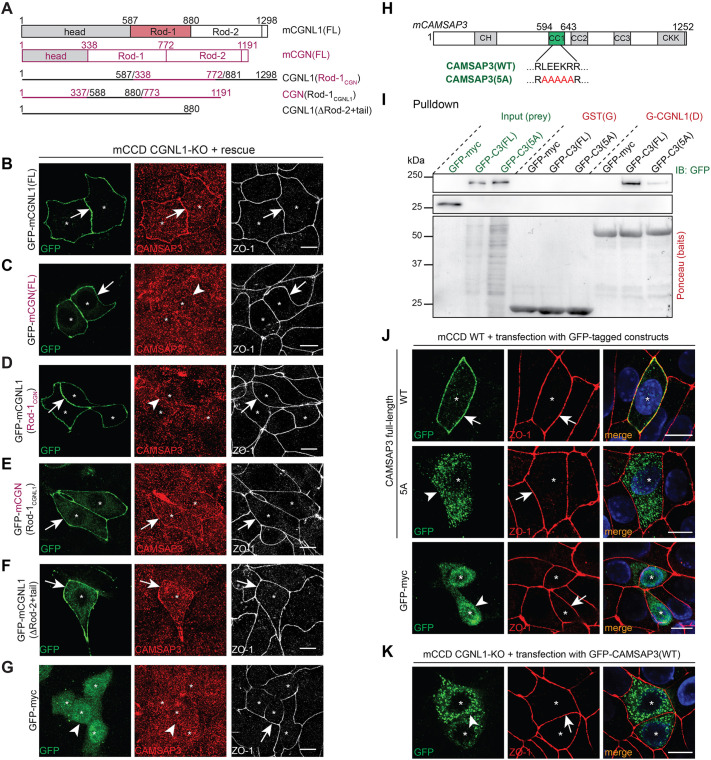
**The Rod-1 domain of CGNL1 is necessary and sufficient for CAMSAP3 junctional recruitment through binding to a 5-amino-acid stretch in the coiled coil region 1 of CAMSAP3.** (A) Scheme of structural domains of mouse CGNL1 (black) and CGN (purple), and of constructs used for rescue experiments shown in B–G. Numbers indicate amino acid boundaries. (B–G) Immunofluorescence microscopy analysis of the localization of endogenous CAMSAP3 (red) at junctions of mCCD CGNL1-KO cells rescued with the indicated GFP-tagged constructs (green, schemes in A). ZO-1 (white) was used as a junctional marker. Asterisks indicate transfected cells. Scale bars: 10 µm. (H) Scheme of CAMSAP3 GFP-tagged constructs used in I and J, with the indicated sequence within the first coiled-coil region of CAMSAP3 (CC1), either WT [CAMSAP3(FL)] or mutated [CAMSAP3(5A)]. Numbers indicate amino acid boundaries. (I) IB analysis, using anti-GFP antibodies, of GST pulldowns using either GST or the CAMSAP3-binding region of CGNL1 (fragment D) as baits, and GFP (negative control), GFP-tagged full-length WT CAMSAP3 [GFP-C3(FL)] or GFP-tagged mutated CAMSAP3 [GFP-C3(5A)] as preys. (J,K) Immunofluorescence microscopy analysis of the localization of exogenous GFP-CAMSAP3 constructs (green) WT and mutant (described in H) in WT mCCD cells (J) and CGNL1-KO cells (K), using ZO-1 (red) as a junctional marker. Asterisks indicate transfected cells. The localization of the 5A mutant and GFP control in CGNL1-KO cells was the same as in WT cells [e.g. cytoplasmic (not shown)]. Arrows and arrowheads indicate detectable and decreased/undetectable labeling, respectively. Blots and images shown in this figure are representative of three repeats. A quantification of B–G is shown in Fig. S6C. Scale bars: 10 µm.

To determine the relevance of the Rod-1 region of CGNL1 in the junctional recruitment of CAMSAP3, we carried out rescue experiments. CGN and CGNL1 are recruited to junctions through the interaction of their N-terminal head sequences with ZO-1, and the coiled-coil Rod domain alone is not localized at junctions (D'Atri et al., 2002; Pulimeno et al., 2011; Vasileva et al., 2022). Thus, we generated chimeric CGN–CGNL1 molecules with switched Rod-1 regions (schemes in Fig. 7A), and we asked whether they could rescue CAMSAP3 localization at junctions in the context of CGNL1-KO mCCD cells (Fig. 7B–G). CAMSAP3 junctional labeling was rescued by full-length CGNL1 (arrows in Fig. 7B; quantification in Fig. S6C). However, neither full-length CGN nor GFP rescued CAMSAP3 junctional labeling, confirming that CGN does not bind to CAMSAP3 nor recruit it to junctions (arrowheads in Fig. 7C,G; quantification in Fig. S6C). Importantly, the chimeric CGNL1 molecule harboring the Rod-1 of CGN also failed to rescue junctional CAMSAP3 (arrowhead in Fig. 7D; quantification in Fig. S6C). In contrast, both the chimeric CGN construct harboring the Rod-1 region of CGNL1 (arrow in Fig. 7E; quantification in Fig. S6C) and truncated CGNL1, lacking the Rod-2 and tail domains, which interacts with non-muscle myosin-2 (Rouaud et al., 2023) (ΔRod2+tail, arrow in Fig. 7F; quantification in Fig. S6C), rescued junctional CAMSAP3. These experiments demonstrate that the Rod-1 region of the coiled-coil domain of CGNL1 is necessary and sufficient, in the context of a chimeric molecule targeted to junctions, for CAMSAP3 recruitment to TJs, whereas interaction of CGNL1 with myosin-2 is not required.

Next, we examined the role of a 5-amino-acid stretch within the coiled-coil 1 (CC1) domain of CAMSAP3 (LEEKR). This stretch is rich in basic and acidic residues and is involved in the localization of CAMSAP3 near the apical membrane of polarized epithelial cells (Noordstra et al., 2016; Toya and Takeichi, 2016). A full-length CAMSAP3 construct where the five residues are mutated to alanine [Fig. 7H, CAMSAP3(5A)] retains its ability to interact with microtubules through the C-terminal CKK domain (Toya et al., 2016). Immunoblot analysis of GST pulldowns showed that the mutant CAMSAP3 prey interacted weakly with the CGNL1 bait, compared to WT CAMSAP3 [Fig. 7I, GFP-C3(5A); quantification in Fig. S6D]. Moreover, when exogenously expressed in mCCD cells, WT CAMSAP3 was almost exclusively detected at junctions (arrow in Fig. 7J, top panel), whereas the 5A mutant was mostly distributed in the cytoplasm (arrowhead in Fig. 7J, middle panel). Importantly, full-length WT CAMSAP3 was not targeted to junctions in CGNL1-KO cells (Fig. 7K), confirming the role of CGNL1 in the junctional recruitment of CAMSAP3, independently of the use of antibodies to label CAMSAP3.

Finally, we examined the role of microtubule network integrity in the junctional and apical localizations of CAMSAP3. To this purpose, we treated mCCD cells with either nocodazole or DMSO (negative control). Nocodazole treatment, but not DMSO, abolished the apical labeling for CAMSAP3 in WT mCCD cells (‘ap.’ arrow and arrowheads in Fig. S7A,B). However, the junctional localization of CAMSAP3 was not affected by nocodazole treatment (‘junct.’ arrows in Fig. S7A).

Together, these experiments indicate that the interaction of the coiled-coil Rod-1 domain of CGNL1 with the 5-residue stretch within the CC1 domain of CAMSAP3 is required for the junctional recruitment of CAMSAP3, and this is independent of microtubule polymerization.

### CAMSAP3 recruitment at junctions is important for cyst morphogenesis but not to organize the PAN of microtubules

The KO of CGNL1 resulted in perturbed cyst morphogenesis for mCCD cells (Fig. 1E–I), and in a dramatic change of the organization of the PAN in Eph4 cells (Fig. 3E–H). However, it is not clear whether these phenotypes are dependent on the loss of junctional CAMSAP3 or on other properties and/or binding partners of CGNL1. To address this question, we generated stable cell lines expressing chimeric constructs of CGNL1 and CGN in the background of Eph4 and mCCD CGNL1-KO cells.

**Fig. 8.**
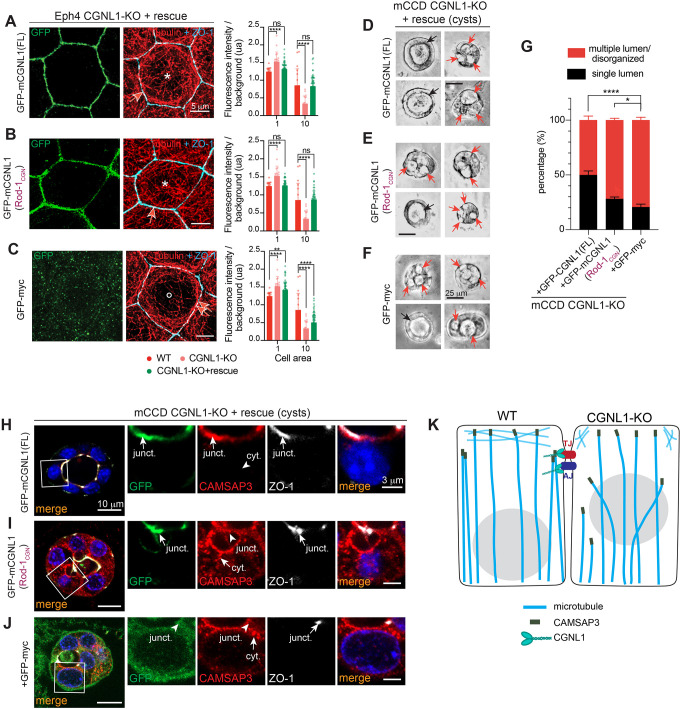
**The Rod1 domain of CGNL1 is necessary for correct epithelial morphogenesis but not for the regulation of the microtubule PAN.** (A–C) Immunofluorescence microscopy analysis (STED) of the planar apical network of microtubules of Eph4 CGNL1-KO cells stably rescued with the indicated GFP-tagged constructs (schemes in Fig. 7A). Microtubules are labeled with tubulin (red); ZO-1 (cyan) is used as a junctional marker. Asterisks and circles indicate homogenous apical microtubule network and absence of microtubules, respectively. Arrows and double arrows indicate normal and increased association of apical microtubules with junctions, respectively. Scale bars: 5 µm. Graphs on the right of images show quantification of labeling intensity for tubulin in the area 1 and 10 of the cell (see representation in Fig. 3G) for WT (red, *n*=18 cells), CGNL1-KO (light red, *n*=20) and CGNL1-KO cells rescued with the respective GFP-tagged construct [green, *n*=56 (A) or 48 (B) or 59 (C)]. Bars represent mean±s.d. for three biological replicates. **P<0.01; ****P<0.0001; ns, not significant (two-way ANOVA with post hoc Sidak's test). (D–F) Phase-contrast images of cysts for mCCD CGNL1-KO cells stably rescued with the indicated GFP-tagged constructs (schemes in Fig. 7A). Black arrows indicate a unique lumen, and red arrows indicate multiple lumens within the same cyst. Scale bars: 25 µm. (G) Quantification of 3D cyst formation of mCCD CGNL1-KO rescue cells as shown in D–F. Percentage of single lumen (black) and multiple lumen (red) are plotted for each cell line. GFP-CGNL1(FL), *n*=290 cysts; GFP-CGNL1(Rod1_CGN), *n*=348 cysts, GFP-myc, *n*=304 cysts. Bars represent mean±s.d. for four biological replicates. *P<0.05; ****P<0.0001 (two-way ANOVA with post hoc Sidak's test). (H–J) Immunofluorescence microscopy analysis of the localization of endogenous CAMSAP3 (red) in cysts of mCCD CGNL1-KO cells rescued with the indicated GFP-tagged constructs (schemes in Fig. 7A). ZO-1 (white) was used as a junctional marker. Magnified images on the right show details of apical and lateral area outlined by boxes. Junctional (junct.) and cytoplasmic (cyt.) localizations are indicated by arrows. Scale bars: 10 µm (main images); 3 µm (magnifications). (K) Scheme showing the organization of microtubules and the localization of CAMSAP3 in WT (left) and CGNL1-KO (right) cells. TJs (red) and AJs (blue) are shown at the apical-lateral borders. The altered organization of apical microtubules, the redistribution of CAMSAP3 from the TJ to the cytoplasm and the altered position of the nucleus are also shown.

The organization of the PAN of microtubules was assessed by quantifying the fluorescence intensity for tubulin in the areas 1 and 10 of the cell according to the previously used segmentation approach (Fig. 3G). Constructs encoding either CGNL1 or a chimeric molecule comprising the Rod1 domain of CGN within the backbone of CGNL1 restored a homogenous PAN of microtubules throughout the apical region (asterisks in Fig. 8A,B; quantification for area 10 on the right) and decreased peri-junctional microtubule bundles (single arrows in Fig. 8A,B; quantification for area 1 on the right). Instead, in cells rescued with GFP alone (negative control) the organization of microtubules was similar to that in CGNL1-KO cells (Fig. 8C). These observations indicate that regulation of the organization of the PAN by CGNL1 is independent of its binding to CAMSAP3 and suggest that the microtubule-binding ability of CGN is sufficient to rescue the phenotype.

Cyst morphogenesis was monitored in rescued mCCD cell lines. The re-expression of full-length CGNL1 significantly increased the percentage of cysts with a single lumen (by about double) compared to the expression of the GFP tag alone (black arrows in Fig. 8D,F; quantification in Fig. 8G). The CGNL1 chimeric construct containing the Rod1 domain of CGN, however, showed a far lower degree of rescue for the phenotype, given that most cysts showed multiple lumens (red arrows in Fig. 8E,F; quantification in Fig. 8G). Immunofluorescence analysis of these cysts confirmed that only the re-expression of full-length CGNL1 rescued the localization of CAMSAP3 at junctions (‘junct.’ arrows and arrowheads in Fig. 8H–J), while eliminating the cytoplasmic localization. Instead CAMSAP3 was cytoplasmic and not junctional in cysts expressing either the chimeric mutant of CGNL1 harboring the Rod-1 of CGN or GFP alone (‘cyt.’ arrows and arrowheads in Fig. 8H–J).

These experiments indicate that the binding of CGNL1 to CAMSAP3 is required for proper lumen formation in cysts, but this interaction is not necessary for the apical organization of microtubules by CGNL1.

## DISCUSSION

Here, we describe new roles for CGNL1 in recruiting the microtubule minus-end binding protein CAMSAP3 to epithelial junctions and in regulating microtubule organization and polarized architecture of epithelial cells. As summarized schematically in Fig. 8K, the KO of CGNL1 results in several phenotypes, including: (1) the loss of CAMSAP3 localization at junctions and its redistribution to the cytoplasm, which we observed in both cultured cells and mouse tissues; (2) the redistribution of microtubules of the planar apical network from a homogeneous apical pattern towards increased accumulation at peri-junctional areas in Eph4 cells; and (3) the altered apical-basal positioning of nuclei in columnar intestinal epithelial in mouse intestine.

Several lines of evidence support the conclusion that CGNL1, and not PLEKHA7, recruits CAMSAP3 to junctions. First, KO of CGNL1, but not of PLEKHA7, resulted in decreased endogenous junctional CAMSAP3 and increased cytoplasmic labeling in both cultured cells (mCCD and Eph4) *in vitro* and mouse epithelia *in vivo*. Second, exogenous CAMSAP3 localized to junctions in WT but not CGNL1-KO cells. Third, GST pulldown experiments showed that CGNL1 interacts much more strongly with CAMSAP3 than PLEKHA7. Fourth, the KO of CGNL1 and not of PLEKHA7 in mice resulted in the loss of basal alignment of nuclei in the intestinal epithelium, reminiscent of the altered epithelial architecture in mice with a functional inactivation of CAMSAP3 (Toya et al., 2016). Our results are at variance with those reported in Meng et al. (2008), where PLEKHA7 was reported to recruit CAMSAP3 to AJs, possibly because of differences in the epithelial cell model and in the depletion approaches. For example, in Caco2 cells CAMSAP3 is associated with junctions only in cells at sparse density, whereas in confluent Caco2 monolayers CAMSAP3 is exclusively apical (Meng et al., 2008; Noordstra et al., 2016). In contrast, in confluent WT mCCD and Eph4 monolayers CAMSAP3 was both junctional and apical. Furthermore, we used KO cells, whereas the studies in Caco2 cells (Meng et al., 2008) were carried out with a siRNA-mediated depletion approach, which could result in different phenotypic outcomes related to the acute depletion of PLEKHA7. Moreover, in Caco2 cells the junctional localization of CAMSAP3 was inhibited by nocodazole (Meng et al., 2008; Noordstra et al., 2016), whereas we found no effect of nocodazole on the junctional localization of CAMSAP3 in mCCD cells. These results suggest that in our cellular models CAMSAP3 is recruited to junctions through strong interaction with CGNL1, independently of microtubules, consistent with the strong *in vitro* interaction between CGNL1 and CAMSAP3. Our results on mouse tissues confirm that the CGNL1-associated pool of CAMSAP3 is physiologically relevant.

The molecular composition and spatial organization of apical junctions is cell specific (Vasileva et al., 2017), and the relative proportions of CGNL1 at TJs versus AJs depends on cell type (Ohnishi et al., 2004). Our results indicate that the TJ-associated pool of CGNL1 is primarily responsible for the recruitment of CAMSAP3 to the AJC. In Eph4 cells, the KO of ZO-1 resulted in a significant decrease in CAMSAP3 junctional labeling, despite the presence of residual AJ-associated CGNL1. Why AJ-associated CGNL1 would not promote the junctional recruitment of CAMSAP3 is not clear, but it could be due to multivalent interactions and/or post-translational modifications of the AJ-associated CGNL1 pool. Importantly, in mCCD cells the KO of PLEKHA7 did not affect the junctional accumulation of either CAMSAP3 or CGNL1, suggesting that in these cells CGNL1 is mostly associated with TJs, and this TJ pool is entirely responsible for the junctional recruitment of CAMSAP3.

We analyzed the behavior of mutant proteins to gain insights into the structure–function relationships of CAMSAP3 and CGNL1. GST pulldown assays showed that the Rod-1 domain of CGNL1 strongly interacted with CAMSAP3 by GST pulldown, and was necessary and sufficient for rescue of CAMSAP3 localization at junctions of CGNL1-KO cells. Instead, although CGNL1 interacts with myosin-2 through its Rod-2 region (Rouaud et al., 2023), this region was not required to rescue CAMSAP3 to junctions. Moreover, mutation of a short sequence in the CC1 region of CAMSAP3, which does not affect the function of CAMSAP3 as it does not prevent binding to microtubules (Toya et al., 2016), abolishes both CAMSAP3 interaction *in vitro* with CGNL1, and its junctional localization. Together, these observations support a model where the Rod-1 region of CGNL1 regulates the junctional localization of CAMSAP3 by directly interacting with the CAMSAP3 coiled-coil region harboring the 5-amino-acid stretch.

In addition to the regulation of CAMSAP3 localization at junctions, we observed that KO of CGNL1 also leads to altered organization of the PAN of microtubules in Eph4 cells, altered cyst morphogenesis of mCCD cells, a more random orientation of apico-basal microtubules and apico-basal repositioning of nuclei in mouse intestinal columnar epithelium. Interestingly, although the Rod-1 region of CGNL1 was required to rescue the cyst morphogenesis phenotype, constructs comprising the Rod-1 region of CGN were also effective in rescuing the planar apical network of microtubules. This suggests that CGN and CGNL1 redundantly control the organization of the planar apical network in Eph4 cells, presumably through direct binding to microtubules (Yano et al., 2013; Mangan et al., 2016; Vasileva and Citi, 2018) but independently of CAMSAP3. Additional studies are required to address this hypothesis, for example by identifying and mutating the microtubule-binding sequences of CGNL1. Given that CAMSAP3-depleted intestinal cells show random microtubule orientation and disordered positioning of nuclei (Noordstra et al., 2016; Toya et al., 2016), CGNL1 could regulate microtubule orientation and apico-basal positioning of nuclei through its ability to recruit CAMSAP3 at junctions. Importantly, because the apical CAMSAP3 localization was maintained in CGNL1-KO cultured cells and tissues, our results suggest that apical CAMSAP3 does not have a critical role in the phenotypes we observe in CGNL1-KO cells. On the other hand, since forced mis-localization of CAMSAP3 perturbs microtubule organization and epithelial architecture in Caco2 cells (Toya et al., 2016), it appears more likely that the redistribution of junctional CAMSAP3 to the cytoplasm in CGNL1-KO epithelial cells plays a key role in the phenotypes of CGNL1-KO cells and tissues. Finally, we do not rule out the possibility that additional interactions of CGNL1, for example with microtubules through the head domain (Vasileva and Citi, 2018), or with GEFs and GAPs for Rho GTPases through the Rod-1 domain (Guillemot et al., 2008a; Rouaud et al., 2020) contribute to the phenotypes described here. It is also important to note that although CGN has also been implicated in organizing the PAN of microtubules in Eph4 cells and regulating cyst morphogenesis (Yano et al., 2013; Mangan et al., 2016), CGN does not interact with CAMSAP3, and CGN-KO mouse tissues do not reveal altered architecture of polarized epithelial cells (Guillemot et al., 2012). This functional difference between CGN and its paralog CGNL1 might in fact depend on the CAMSAP-3-binding properties of CGNL1.

In summary, we provide evidence for a role of CGNL1 in the junctional recruitment of CAMSAP3, the organization of microtubules, and morphogenesis and architecture of epithelial cells *in vitro* and *in vivo*. Since both CGN and CGNL1 are also involved in tethering specific myosin-2 isoforms to apical junctions (Vasileva et al., 2020 preprint; Rouaud et al., 2023), our observations highlight a central role of CGN and CGNL1 in orchestrating the organization of both actomyosin and microtubule cytoskeletons.

## MATERIALS AND METHODS

### Cell culture and transfection

Culture conditions for mouse cortical collecting duct cells (mCCD Tet-on), mouse mammary epithelial cells (Eph4) and human embryonic kidney epithelial cells (HEK293T) were described previously (Vasileva et al., 2022). CGNL1-KO and CGN-KO mCCD and Eph4 cells and PLEKHA7-KO mCCD cells were described previously (Shah et al., 2018; Vasileva et al., 2022).

To generate rescue stable cell lines in Eph4 and mCCD CGNL1-KO, cells were transfected with relevant pTRE2hyg plasmids (JetOptimus, Polyplus). At 48 h post transfection, single cells were sorted into 96-well tissue culture plates (using a Beckman Coulter MoFlo Astrios sorter, Flow Cytometry Service, University of Geneva Medical School). Single clones were then amplified and screened for the rescue using immunoblot and immunofluorescence analyses. Selected clones were cultivated in DMEM with hygromycin (200 µg/ml; InvivoGen, ant-hg-2). In addition, doxycycline (4 µg/ml; Sigma-Aldrich, D9891) was added in mCCD medium to mediate expression of the transgene. All cell lines were regularly tested for mycoplasma contamination.

Cells were seeded either on 12-mm #1.5 round glass coverslips in 24-well plates, or on 22×22 mm #1.5 square glass coverslips in six-well plates (STED) or on 6.5-mm 0.4-µm-pore polyester 24-well tissue culture inserts (Transwell filters; Corning Costar; #3470). Transfection (JetOptimus, Polyplus) was performed 1 day after seeding, on cells at 60–80% confluence, and following the manufacturer's guidelines. Cells were fixed and processed for immunofluorescence 48–72 h after transfection.

To depolymerize microtubules, cells were treated with 10 µM nocodazole (Sigma-Aldrich; SML1665) for 2 h at 37°C before being fixed and processed for immunofluorescence. Dimethyl sulfoxide (DMSO, maximal final concentration of 0.1%) treatment was used as negative control.

Cysts of mCCD cells were cultured following the protocol of Debnath et al. (2003). Glass coverslips in a 24-well plate were coated with 40 µl of Matrigel (354230, BD Biosciences) and allowed to solidify for 30 min at 37°C. Cells were trypsinized, and resuspended in normal culture medium to obtain a single-cell suspension. Cells were diluted to 60,000 cells/ml and mixed in a 1:1 ratio with assay medium [SMEM; M8167, Sigma-Aldrich), 4% Matrigel and 10 ng/ml epidermal growth factor (EGF)], and 400 µl was plated per well. mCCD cysts were grown up to 14 days, replacing medium with fresh assay medium every 4 days.

### Generation of CGNL1-KO mice

CGN-KO mice were obtained as described previously (Guillemot et al., 2012). A CGNL1 targeting vector (E1pTV) was designed to insert two LoxP sites within the mouse CGNL1 locus, one upstream of exon 1 and two downstream of exon 2 (SpeI, SalI), as well as two FRT sites flanking the Neo resistance cassette (SalI) (Fig. S3A, targeting vector). Exon 1 contains the putative ATG, and exon 1 and exon 2 code for the head domain, which is functionally critical for the junctional recruitment of CGNL1. The targeting vector was linearized by NheI digestion prior to transfection into hybrid embryonic stem cells (C57BL/6×129/SvEv). Genomic DNA samples from neo-resistant ESC clones (*n*=288) were analyzed by Southern blot analysis, using 3′ probe labeled by the DIG High Prime kit (Roche, Switzerland). Transfection of ESCs, selection of neo-resistant clones, preparation of lysates for genotyping and subsequent generation of the heterozygous F1 mouse was carried out by InGenious Targeting Laboratory (Stony Brook, NY, USA). Positive ESC clones were microinjected into Balb/c blastocysts. Resulting chimeras with a high percentage agouti coat color were mated to wild-type C57BL/6 mice to generate F1 heterozygous offspring. DNA from F1s was genotyped by PCR using primer set NEO3+ A2 (NEO3: 5′-GTGGTTCTAAGTACTGTGGTTTCCAAA-3′; A2: 5′-GTAATAGCATGTGTGCCCATCTGAAA-3′), with NEO3 annealing inside the NEO cassette and A2 annealing 3′ to the short homology arm, outside the region used to create the targeting construct (amplicon is 2.5 kb in length). Targeting vector integration and the presence of LoxP sites was confirmed by PCR, by sequencing and by Southern blotting of NheI-digested genomic DNA with a probe (PB1/2, 726 bp, generated with primers PB1 5′-AGTCACTCCCTTTGTTGCTTCTAG-3′ and PB2 5′-AGGGAAGGAGCACACAGGGTAAC-3′) against the 3′ external region. Heterozygous mice containing one targeted CGNL1 allele were crossed with MeuCre40 mice (Guillemot et al., 2012), to obtain mosaic mice carrying either targeted or allele or allele with deleted exon 1 and exon 2 (through Cre-mediated deletion). These mice were then crossed to WT C57BL/6 to eliminate the Cre allele. To eliminate the neo cassette, we carried out a final third crossing between heterozygous mice, carrying one WT and one allele with deleted exon1 and exon2 and the NEO cassette, and mice carrying the FLP flippase (obtained from ITL, Stony Brook, NY) and further back-crossed to obtain heterozygous C57BL/6 mice with a homogeneous genetic background, carrying a deletion exon 1 and exon 2 of CGNL1. For genotyping, genomic DNA was extracted using Thermo Fisher Scientific Phire Tissue Direct PCR Master Mix and analyzed by PCR using the primers indicated in Fig. S3A: (A) 5′-TGTAATTAGTATATGCTACTAGT-3′ (forward); (F) 5′-GTCTCAGCTGTCTCTCAAC-3′ (reverse) and (C) 5′-CCTGCTTATCTTGGGAGACTT-3′ (forward). Phire Hot Start II DNA polymerase was used for amplification and the products were loaded onto a 1.5% agarose gel and visualized using EZ-Vision Blue light DNA Dye.

Mice were housed with 12 h light–dark cycles with *ad libitum* access to standard chow and water. They were cared for and treated in accordance with the guidelines of the Direction Générale de la Santé, State of Geneva (license numbers GE/1027/3853/3, GE/31.1.1010/1800/I, GE/67/15, GE/9/18, GE/68/17, GE/133/20). Mice were back-crossed with the WT C57BL/6 strain for 10 generations before analysis. Mice with homozygous mutation of the CGNL1 allele were viable, and showed no apparent gross anatomical abnormality, but were sterile. Crossing of heterozygous CGNL1 mice gave progeny in expected mendelian ratios (Table S2). The detailed phenotypic characterization of CGNL1-KO mice will be reported elsewhere.

### Generation of PLEKHA7-KO mice

The PLEKHA7-KO mice were generated by pronuclear injection as previously described (Hogan et al., 1994). The gene was targeted using the following CRISPR sgRNA sequences: 5′-TCGAGAGACTGTCCTCGTCG-3′ and 5′-CAGCCCCGTGCGGACGCCTC-3′, which were cloned into the Cas9-encoding pX458 vector (Addgene #48138). These plasmids were injected in B6D2F1 one-cell embryos and, the resulting injected embryos were then transferred into CD1 pseudo-pregnant mothers. After birth, F0 founders containing mutations in the PLEKHA7 gene were identified through genomic DNA sequencing. Mice with homozygous mutation of the PLEKHA7 allele were viable, showed no apparent gross anatomical abnormality, and were fertile. Crossing of heterozygous PLEKHA7 mice gave progeny in expected mendelian ratios (Table S2). The phenotypic characterization of PLEKHA7-KO mice will be described elsewhere.

### Antibodies

The primary antibodies (see also Table S1 for host species, antigen, source and identifier) were used at the following dilutions for immunoblotting (IB), immunofluorescence microscopy (IF) and immunohistochemistry (IHC): rabbit CAMSAP3 [IB: 1:2000; IF: 1:200; IHC: 1:200; raised against mouse CAMSAP3, specificity validation shown in Meng et al. (2008) and (Tanaka et al. (2012)]; guinea pig CAMSAP3 (SZC112; IHC: 1:50); rabbit paracingulin (20893; IB: 1:10,000); rabbit paracingulin (821; IF: 1:1000); mouse paracingulin (IF: 1:1000); rabbit cingulin (C532; IF: 1:1000; IB: 1:2000); mouse cingulin (22BD5A1; IF: 1:500); rabbit PLEKHA7 (Rb30388; IB: 1:5000, IF: 1:1000); guinea pig PLEKHA7 (GP2737; IF: 1:300, IHC: 1:300); mouse E-cadherin (610181; IB: 1:5000, IF: 1:1000); rat ZO-1 (R40.76; IF: 1:100; IHC: 1:100); mouse β-tubulin (32-2600; IB: 1:3500); rabbit α-tubulin (ab18251; IF: 1:500); guinea pig α- and β-tubulin (scFv-S11B and scFv-F2C; IF: 1:400); rabbit laminin (L9393; IHC: 1:200); rabbit MACF1 (ab117418; IF: 1:400); rabbit polyE (AG-25B-0030; IHC: 1:500); mouse GFP (11814460001; IB: 1:2000; IF: 1:200) and mouse HA (32-6700; IF: 1:150). For U-ExM, primary antibodies were concentrated two times compared to conventional IF.

Secondary antibodies for immunofluorescence were from Jackson ImmunoResearch and diluted at 1:300 (1:1000 for cryostat tissue sections): anti-mouse-IgG (715-546-151), anti-rabbit-IgG (711-545-152), anti-rat-IgG (712-546-153) and anti-guinea pig-IgG (706-546-148) conjugated to Alexa Fluor 488; anti-mouse-IgG (715-165-151), anti-rabbit-IgG (711-165-152) and anti-rat-IgG (712-166-153) conjugated to Cy3; anti-guinea pig (706-605-148), anti-rabbit-IgG (711-605-152) conjugated to Alexa Fluor 647; anti-rat-IgG (712-175-153) and anti-mouse-IgG (715-605-151) conjugated to Cy5. For STED microscopy, secondary antibodies from Abberior were used at 1:300: anti-rabbit-IgG conjugated to STAR 580 (ST580-1007) and anti-rat-IgG conjugated to STAR RED (STRED-1002).

The guinea pig polyclonal antibody targeting CAMSAP3 (SZC112; IB: 1:1000, IHC: 1:100) was generated using as immunogen a purified mouse CAMSAP3 peptide, the same as was used for the production of the rabbit polyclonal anti-CAMSAP3 described in Meng et al. (2008) (NP_081447.2; aa 1085-1098: SRLPGSRERDWENG; Polyclonal Antibody Production, Eurogentec).

### Plasmids

Constructs that were described previously include GST-tagged fragments of CGNL1 in pGEX4T1 (Guillemot et al., 2008b) and GFP-tagged full-length (FL) mouse ZO-1 (S2474) (Rouaud et al., 2023) (Table S1).

GFP-tagged full-length and truncated constructs of mouse CAMSAP3 [FL: 1-1252 (S2554); ΔCH: 594-1252 (S2558); ΔCCs: 1-593+948-1252 (S2670); ΔCKK: 1-1111 (S2671); CCs: 594-947 (S2672)] were obtained by PCR and subcloning into pcDNA3.1(-) plasmid using the InFusion cloning technique, previously modified to contain N-terminal GFP (pcDNA3.1-GFP) (Paschoud et al., 2011).

The following WT and mutant plasmids were obtained by PCR with appropriate oligonucleotides on full-length cDNAs and subsequently cloned by infusion cloning technique into pCDNA3.1-GFP: (1) GFP-tagged mutant construct of mouse CAMSAP3 (5A; amino acids 608–612 mutated to alanine residues) (S2797); (2) GFP-tagged constructs of FL mouse CGNL1 and mouse CGN (S2799 and S2801, respectively); (3) GFP-tagged chimeric constructs of mouse CGNL1 and mouse CGN (S2800 and S2802, respectively); (4) GFP-tagged truncated construct of mouse CGNL1(Δrod2+tail: 1–880) (S2815). The following plasmids were obtained by PCR with appropriate oligonucleotides on full-length cDNAs and subsequently cloned by infusion cloning technique into pTRE2hyg, previously modified to contain N-terminal GFP (pTRE2hyg-GFP, S1210): (1) GFP-tagged construct of FL mouse CGNL1 (S2875); (2) GFP-tagged chimeric construct of mouse CGNL1 (S2876). GST-tagged truncated mouse CAMSAP3 (CC1+2: 600–729) was obtained by PCR on full-length mouse CAMSAP3 with appropriate oligonucleotides and cloned into pGEX4T1 (EcoRI-NotI) (S2796). GST-tagged truncated human PLEKHA7 (351–820) was obtained by PCR on full-length human PLEKHA7 with appropriate oligonucleotides and cloned into pGEX4T1 (BamHI-XhoI) (S1193). Constructs of C-terminal HA-tagged full-length canis CGNL1 was obtained by amplification of full-length *Canis* CGNL1 with appropriate oligonucleotides, and subsequent cloning into pcDNA3.1(-) (BamHI-NotI) (S2432). All constructs were validated by sequencing (Microsynth, Switzerland).

### Conventional, ultra-expansion and STED immunofluorescence microscopy

For conventional confocal microscopy or STED immunofluorescence microscopy, cells on coverslips were washed once with room temperature (RT) phosphate-buffered saline (PBS) and then fixed with cold methanol (−80°C) for 8 min at −20°C. After three PBS washes, cells were blocked 30 min in PBS with 2% bovine serum albumin (BSA). Primary antibodies diluted in blocking buffer were incubated for either 1 h at RT or 16 h at 4°C. After three PBS washes, secondary antibodies and 1 µg/ml DAPI (AppliChem, A4099) diluted in blocking buffer were added, and cells incubated for 30 min at 37°C, and finally washed with PBS (three times). Mounting medium was Fluoromount-G (0100-01, SouthernBiotech) for conventional confocal microscopy and ProLong Gold (P10144, Thermo Fisher Scientific) for STED microscopy.

For immunofluorescence of cells grown on Transwell inserts, we used the method described previously (Sluysmans et al., 2021). Filters were placed on glass slides, cells facing up, mounted with Fluoromount-G and covered by a glass coverslip.

The proximity ligation assay (PLA) (DU092101, Sigma-Aldrich) was performed according to the manufacturer's protocol.

Expansion microscopy (U-ExM) was carried out as described in Gambarotto et al. (2019). Gels were mounted on 24-mm round 1.5H precision coverslips (0117640, Marienfeld) coated with poly-D-lysine (A3890401, GIBCO) for imaging.

### Mouse tissue immunohistochemistry

Tissues were obtained from mice euthanized by sodium pentobarbitone administration, as recommended by OSAV (Swiss Federal Office for Food Security and Veterinary Affairs) and approved by the above-mentioned Animal Experimentation Permits, and were included in OCT medium and snap-frozen in liquid nitrogen-cooled isopentane. For conventional microscopy, frozen sections (5–10 µm) were air-dried, fixed with acetone at −20°C for 20 min, and rehydrated in PBS (10 min wash, three times). After 30 min of blocking in PBS with 1% donkey serum, sections were incubated with primary antibodies (overnight at 4°C) and secondary antibodies (1 h at RT) diluted in PBS with 1% BSA, 1% donkey serum and 0.3% Triton X-100, each followed by three washes in PBS, and were finally mounted with Fluoromount-G and covered by a glass coverslip. For U-ExM, tissue sections adhering to coverslips were included in gels, expanded and processed as described above.

### Image acquisition

Slides for conventional confocal microscopy were imaged on a Zeiss LSM800 confocal microscope using a 63×/1.4 NA oil immersion objective.

STED imaging was performed on a Leica TCS SP8 STED 3X using an HC PL Apo 93×/1.3 glycerol immersion objective. Abberior Star 580 was imaged with a pulsed laser at 560 nm, and excitation of Abberior Star Red was performed at 640 nm. The depletion laser for both colors was a Katana 775 nm pulsed laser. Generation of deconvolved images was done with the Lightening mode, adaptive as ‘Strategy’ and ProLong Gold as ‘Mounting medium’.

For U-ExM, image acquisition was performed on an inverted Leica TCS SP8 microscope using a HC PL APO 63x/1.4 NA oil immersion objective. Generation of deconvolved images was done with the Lightening mode at maximum resolution, adaptive as ‘Strategy’ and water as ‘Mounting medium’.

Unless otherwise stated, scale bars correspond to 10 µm. Scale bars of U-ExM samples are indicated at the expanded size (not rescaled). The average expansion factor was calculated by dividing the expanded size by the original size of the coverslip (4.2×).

### Quantifications

For the quantification of junctional immunofluorescent signal, pixel intensity was measured in the selected junctional area using the polyhedral tool of Fiji/ImageJ, and the averaged background signal of the channel was subtracted. The relative intensity of junctional signal is expressed as a ratio between the signal of the protein of interest and an internal junctional reference (PLEKHA7, CGNL1 or ZO-1). Between 5 and 20 junctional segments of each phenotype were analyzed for each experiment, each segment being used as a replicate.

For the quantification of the ratio of cytoplasmic to total CAMSAP3 in polarized mCCD cells, the pixel intensity of CAMSAP3 was measured in the cytoplasm and in the entire cell area (*z*-lines) using the polyhedral tool of Fiji/ImageJ. Relative intensity of CAMSAP3 signal was expressed as a ratio between the signal of cytoplasmic CAMSAP3 and the signal of total CAMSAP3. At least three *z*-lines for each cell type were analyzed for each experiment, each *z*-line being used as a replicate.

For the quantification of cyst formation, the number of cysts with single or multiple (≥2) lumen were counted on phase contrast images. Between 200 and 340 cysts were analyzed for each phenotype from three different experiments.

For the quantification of apical tubulin intensity in Eph4 cells, cells were individually selected with their junction using the polyhedral tool of Fiji/ImageJ, and subareas were obtained by scaling down the area repeatedly by 10% (10 areas are obtained for each cell). Pixel intensity was measured in composite selections using the exclusive or (XOR) operation on two adjacent areas. Intensities were then divided by the background signal of the image. Between 8 and 24 cells were analyzed per experiment, each cell being used as a replicate.

For the quantification of CAMSAP3 constructs bound to GST-tagged CGNL1, the chemiluminescence signal intensity of GFP [for GFP tagged CAMSAP3(FL) or CAMSAP3(CC1mut)] was determined using Fiji/ImageJ and normalized to the input. Quantification was performed on data from three separate experiments.

For the quantification of protein levels in mCCD and Eph4 lysates, the chemiluminescence signal intensity of CGNL1, CGN and CAMSAP3 was determined using Fiji/ImageJ and normalized to the tubulin input. Quantification was performed on data from three separate experiments.

For the quantification of colocalization between junctional marker and CAMSAP3 (Pearson's correlation coefficients), colocalization was determined using the Colocalization Threshold plug-in in FIJI software. Autothresholding from the Costes method was applied (images with ‘Pearson's below threshold’ superior to 0.1 were not considered). Several areas (selected with the polyhedral tool of Fiji/ImageJ) from two to five images from three different experiments were used, each area being used as replicate.

For the quantification of the number of microtubules anchored at junctions, each image was carefully analyzed for microtubule ends capped with CAMSAP3 signal within 0.2 µm of junctions (as defined by ZO-1 labeling) and counted. Those numbers were then divided by the length of the junction being analyzed, measured using the StraightLine tool of Fiji/ImageJ. Around 10 junctional segments were analyzed for each experiment, each segment being used as a replicate.

For analysis of polarity defects in intestinal epithelial cells, the distance between the basal lamina (marked with laminin) for the cell height or the middle of the nucleus for the nucleus-apex distance and the apical surface (marked with the apical junctional marker PLEKHA7) was measured using the StraightLine tool of Fiji/ImageJ. At least 10 distances were measured per experiment, using each distance as a replicate.

For the analysis of microtubule directionality in intestinal epithelial cells, an arbitrary line was traced along the apico-basal axis of the intestinal cell, and the angle of the microtubules that crossed the line were measured using the angle tool of Fiji/ImageJ (Toya et al., 2016). The occurrence for each angle was plotted and fitted to a Gaussian curve. The experiment was performed twice, analyzing at least 98 crossing points per experiment.

A color code was used for different experimental repeats for all graphs when showing individual data points.

### Cell and tissue lysates

Cell lysates were obtained as described previously (Sluysmans et al., 2021) with the following modifications: SDS-PAGE was carried out at RT, and transfer onto nitrocellulose membrane was carried out at 85 V for 120 min. Blocking was realized with Tris-buffered saline (TBS) with 0.1% Tween-20 and 5% low-fat milk, and incubation with primary and secondary antibodies in the same buffer containing only 3% low-fat milk.

Chemiluminescence (ECL) was detected using Odyssey Imager (LI-COR) or Amersham ImageQuant 800 (Cytiva). Numbers on the left of immunoblots correspond to sizes in kilodaltons (kDa) of prestained markers. Uncropped blots are shown in Fig. S8.

### Recombinant protein production and GST pulldowns

Production of GST-fused proteins and GST pulldowns were carried out as described in Sluysmans et al. (2021). Prey proteins were expressed in HEK293T cells; as such, we cannot exclude that contaminating proteins from the lysates might affect the outcome of the pulldown; however, due to the high overexpression of prey proteins, it is unlikely that contaminants are present in sufficiently high concentrations to affect the results.

### Immunoprecipitation

Immunoprecipitations were carried out as described previously (Sluysmans et al., 2021), with the following modifications: after incubation of the pellet with SDS buffer, the resultant supernatant was mixed with the cytoskeleton soluble fraction to obtain the total cell lysate.

### Statistical analysis

Data processing and analysis were performed using GraphPad Prism. All experiments were carried out at least twice, when applicable on multiple clonal lines. Statistical significance of quantitative data was determined by either unpaired two-tailed Student's *t*-test (when comparing two sets of data) or ordinary one-way or two-way ANOVA with post hoc Dunnett's (to compare every mean to control mean) or Sidak's test (for multiple comparisons), (ns, not significant, *P*>0.05; significant, *P≤0.05, **P≤0.01, ***P≤0.001 and ****P≤0.0001). All graphs are represented as mean±s.d.

## Supplementary Material

10.1242/joces.260745_sup1 Supplementary information
