# Supplementary material for: Paracingulin recruits CAMSAP3 to tight junctions and regulates microtubule and polarized epithelial cell organization
Source: J Cell Sci. 2023 May 15;137(5):jcs260745. doi: 10.1242/jcs.260745 (PMC10184829; doi:10.1242/jcs.260745)
Supplement: Supplementary information [file joces-137-260745-s1.pdf]

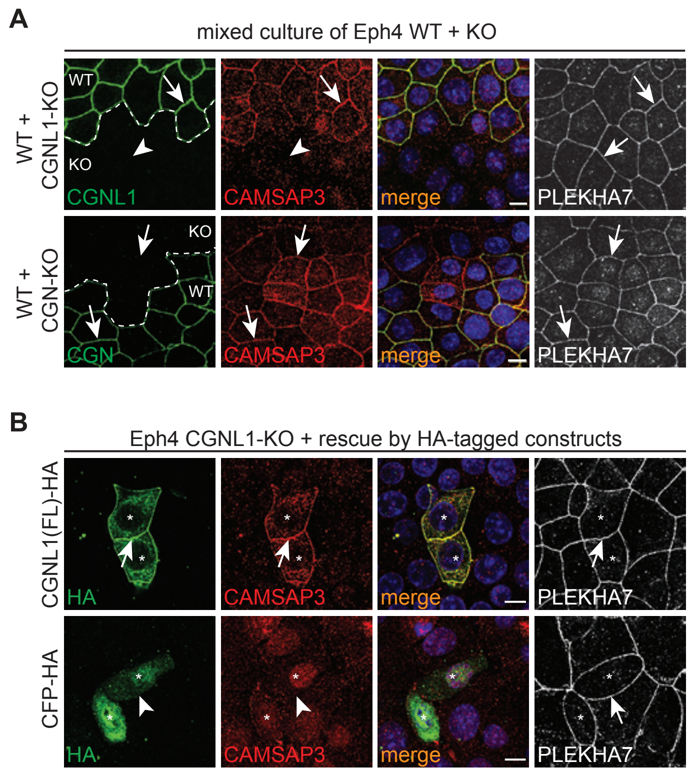

Fig. S1. CGNL1 is responsible for the junctional recruitment of CAMSAP3 in Eph4 cells.(A) IF microscopy analysis of the localization of CAMSAP3 (red) in mixed cultures of WT and CGNL1 or CGN-KO Eph4 cells, using PLEKHA7 (white) as a junctional marker. CGN and CGNL1 stainings (green) are used to distinguish WT from KO cells (white dashed line).

(B) IF microscopy analysis of the localization of CAMSAP3 (red) in CGNL1-KO Eph4 cells transfected with HA-tagged CGNL1 full length (FL) or CFP-HA tag alone (green). PLEKHA7 (white) is used as a junctional marker.

White arrows and arrowheads indicate detectable and undetectable junctional staining respectively. Asterisks indicate transfected cells. Scale bars = 10 μm.

Journal of Cell Science • Supplementary information

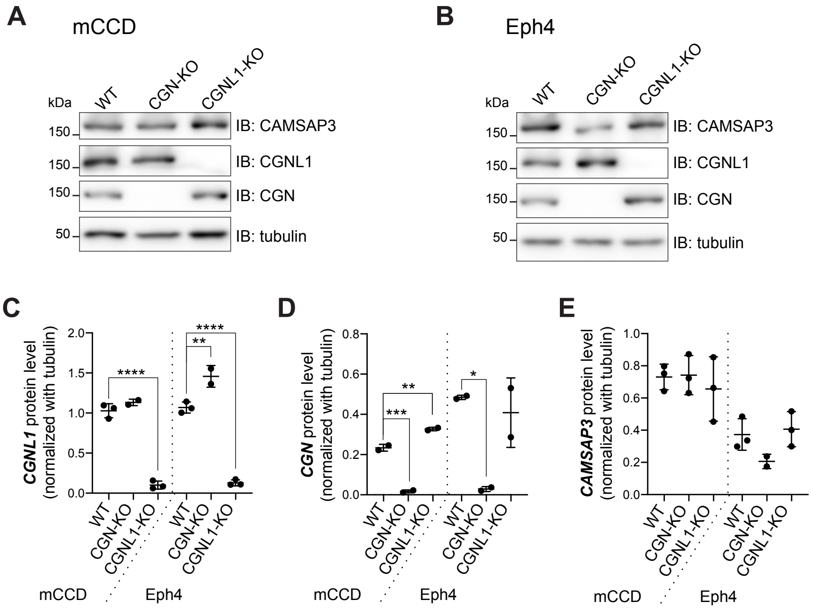

Fig. S2. CAMSAP3 protein expression is not altered by the KO of either CGNL1 or CGN in mCCD and Eph4 cells.

(A-B) Representative Immunoblot (IB) analysis of the expression of CAMSAP3, CGNL1, CGN and tubu-lin from mCCD WT, CGN-KO and CGNL1-KO lysates (A) and from Eph4 WT, CGN-KO and CGNL1-KO lysates (B). Numbers indicate migration of pre-stained size markers. (C-E) Quantification of the expression of CGNL1 (C), CGN (D) and CAMSAP3 (E), normalized with the expression of tubulin. One-way ANOVA with post hoc Dunnett's test (*$p < 0.1$, **$p < 0.01$, ***$p < 0.001$, ****$p < 0.0001$) (n = 2-3).

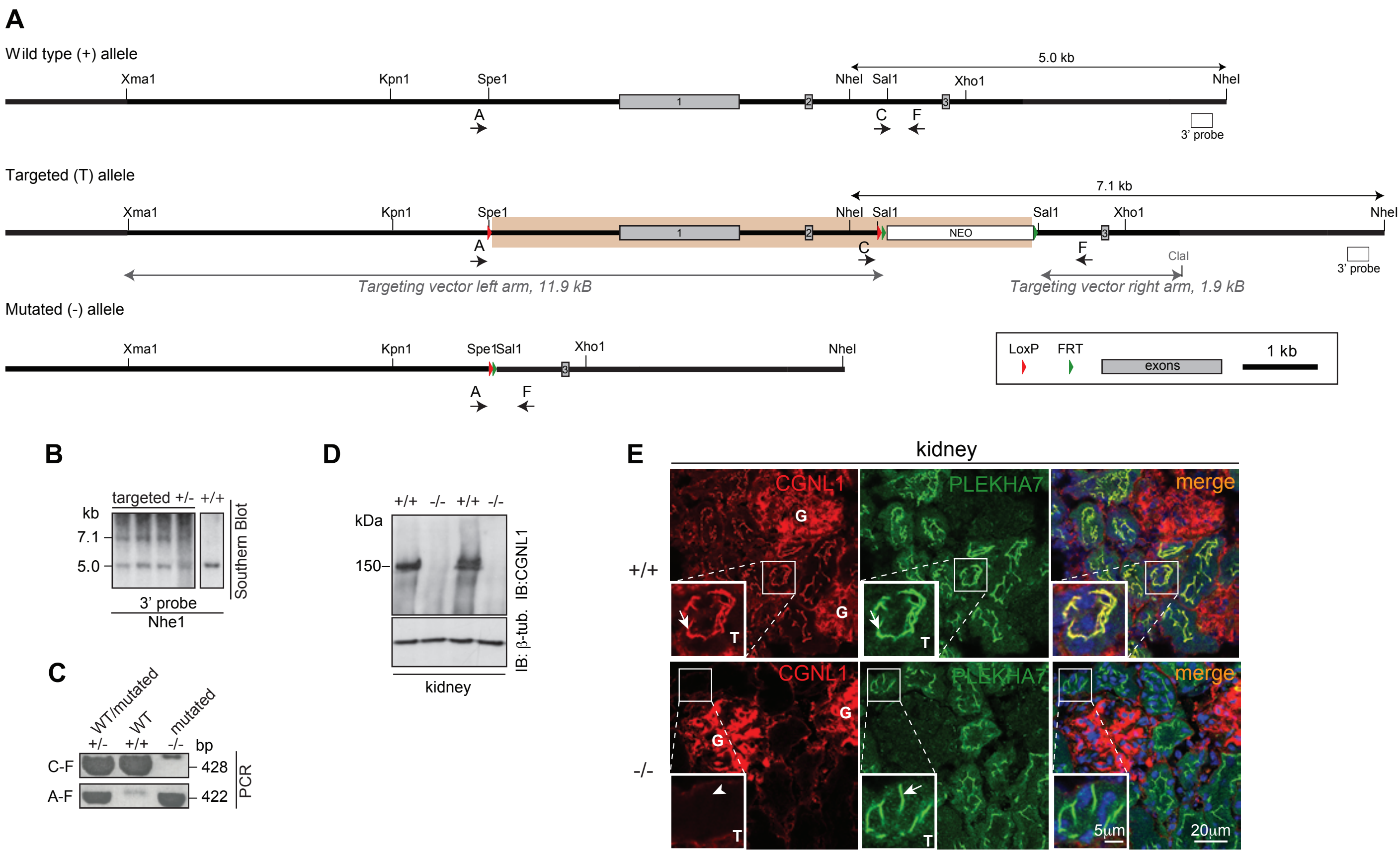

Fig. S3. Generation of CGNL1-KO mice.

(A) Schematic diagrams representing the wild-type allele (+), the targeting vector, the targeted (T) allele, and the mutated (−) allele. The position of the 5′ and 3′ probes, the Neo probe, and the PCR primers (A, C, F) used for genotyping are indicated below the diagrams. Exons are indicated by boxed numbers. Key restriction sites as well as position and size of fragments generated by digestion are indicated above the diagrams. The targeting vector comprises a 11.9 kb left arm containing two LoxP (red triangles) sites upstream of exon 1 (cloned into SpeI site) and upstream of exon 2 (cloned into Sal1 site), respectively, and a 1.9 kb right arm, comprising exon 3. A neomycin resistance cassette (NEO) is cloned between exons 2 and 3 and flanked by FRT (green triangles) sites. Cre-mediated recombination between the first LoxP and the second FRT sites results in the formation of the mutated (−) allele.

Journal of Cell Science • Supplementary information

(B) Southern blot analysis of NheI-digested genomic DNA with the 3′ probe results in a 5 kb fragment in the wild-type allele, and a 7.1 kb fragment in the targeted allele, due to the insertion of the Neo cassette.
(C) PCR amplification of genomic DNA from ear clippings using primer sets ‘C–F’ generates a fragment of 428 bp in the WT allele, and no product in the mutated allele. PCR amplification using primer sets A–F generates a fragment of 422 bp in the mutated allele, and no product in the WT allele.
(D) Immunoblot analysis of the expression of CGNL1 in kidney tissues of WT (+/+) or CGNL1 mutated homozy-gous (-/-) mice. β-tubulin is used as a loading control. Numbers on the left indicate migration of pre-stained size markers.
(E) Immunofluorescence analysis of the localization of CGNL1 (red) in kidney tissues from WT (+/+) or homozy-gous (-/-) mice. Insets on the bottom left of images show high magnification details of areas outlined in square boxes. “T” indicates tubules and “G” indicates glomerulus that retain red labeling in homozygous mice due to secondary antibody cross-reaction with blood. Scale bars are 20 and 5 μm in low and high magnification, respectively.

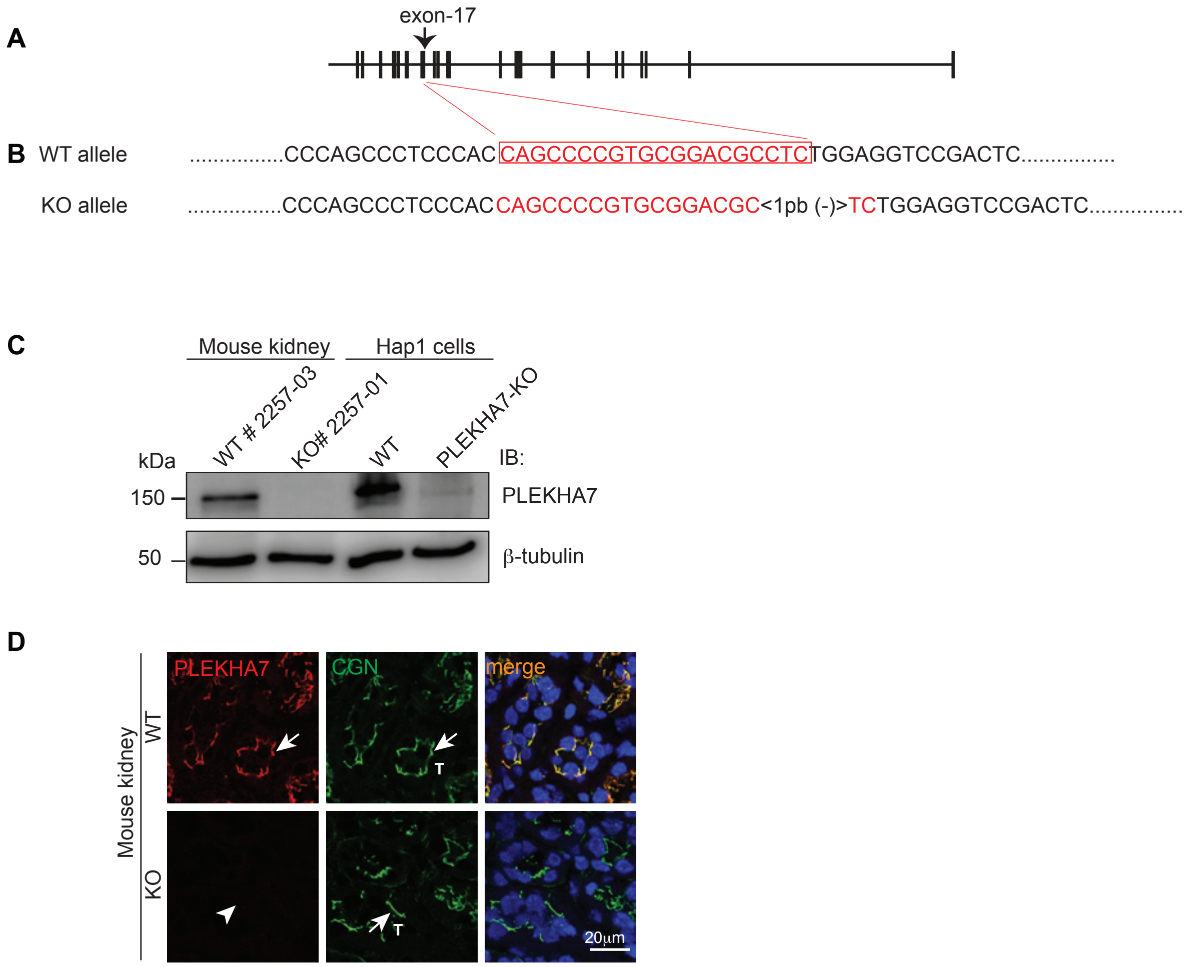

Fig. S4. Generation of PLEKHA7-KO mice.

(A) Scheme of mouse PLEKHA7 gene with exons (vertical bars) and introns (spaces).

(B) WT alleles sequence, guide RNA target sequence (red) and 1 bp deletion (”-”) in the targeted allele, determined by genomic sequencing.

(C-D) Phenotypic characterization of PLEKHA7-KO mouse line either by immunoblotting (IB) (C) or immunofluo-rescence (IF) (D). IB was carried out with rabbit 30388 rabbit anti-PLEKHA7, using lysates of WT and PLE-KHA7-KO Hap1 cells as positive and negative controls, respectively (Shah et al, 2018). Numbers on the left of IB indicate apparent size in kDa, based on the migration of prestained molecular weight markers. IF was carried out with anti-PLEKHA7 (red, guinea pig antibody) and anti-CGN (green, mouse antibody), this latter used as reference junctional marker. Nuclei are stained with DAPI in merge images. “T” indicates tubular epithe-lial cells. Arrows indicate junctional localization, arrowheads indicate undetectable/decreased labeling. Scale bar = 20 μm.

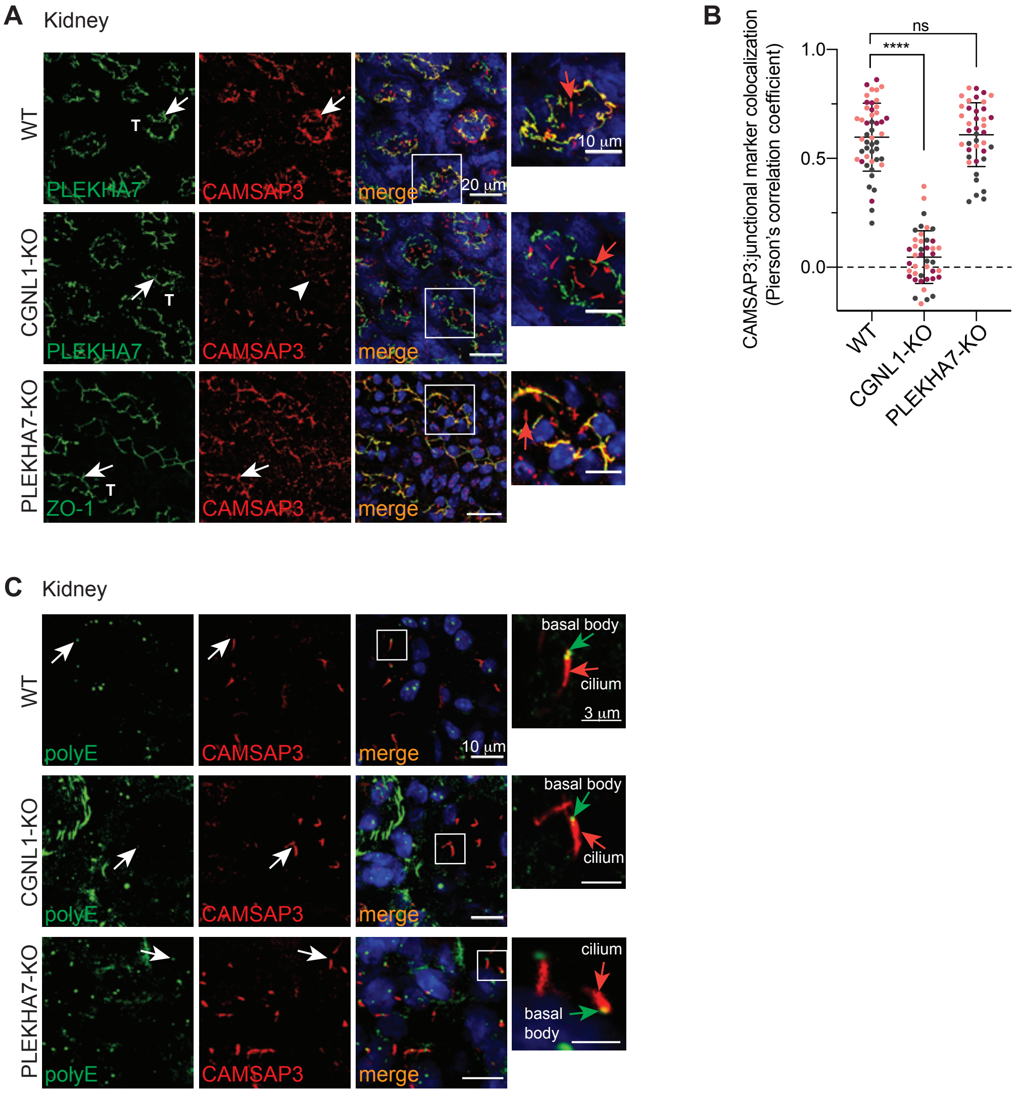

Fig. S5. The KO of CGNL1 alters the junctional, but not cilia, localization of CAMSAP3 in kidney tubular cells.

(A) IF microscopy analysis of the localization of CAMSAP3 (red) in kidney tissue sections from mice either WT or KO for CGNL1 or PLEKHA7. PLEKHA7 or ZO-1 (green) are used as junctional markers. Insets on the right of images show high magnification details of areas outlined in square boxes. White arrows and arrowheads indicate detectable and decreased/undetectable junctional labeling, respective-ly. Red arrows in insets point to CAMSAP3 staining of ciliary-like structures. Scale bars are 20 and 10 µm in low and high magnification, respectively.

(B) Quantification of the colocalization between CAMSAP3 and PLEKHA7 (for WT and CGNL1-KO kidney sections) and ZO-1 (for PLEKHA7-KO kidney sections) using Pierson's correlation coefficient. One-way ANOVA with post hoc Dunnett's test (****p < 0.0001, ns) (*n* = 40-48 areas).

(C) IF microscopy analysis of the localization of CAMSAP3 (red) in kidney tissue section of WT, CGNL1-KO or PLEKHA7-KO mice, using polyglutamylated tubulin (polyE, green), a tubulin PTM, as a basal body marker. Insets on the right of images show high magnification details of areas outlined in square boxes. CAMSAP3 decorates the cilia (red arrows, cilium) that expands from the basal body (green arrows, basal body). Scale bars are 10 and 3 µm in low and high magnification, respectively.

Journal of Cell Science • Supplementary information

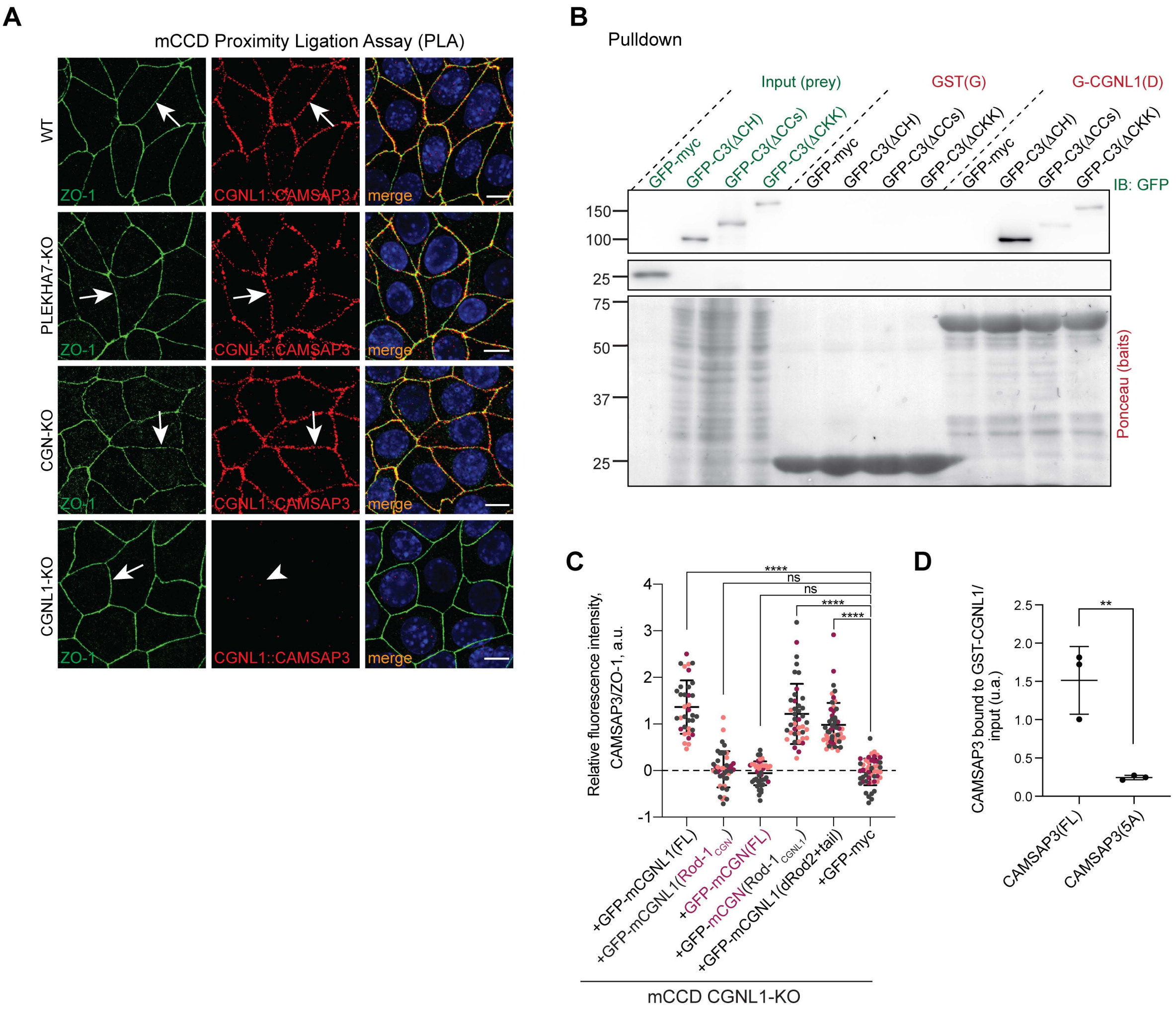

Fig. S6. CGNL1 and CAMSAP3 are in close proximity in cells and directly interact *in vitro* through their coiled-coil domains.

(A) Proximity ligation assay (PLA) between CGNL1 and CAMSAP3 in mCCD WT, CGNL1, CGN or PLE-KHA7-KO mCCD cells (red). The tight junction protein ZO-1 (green) is used as a junctional marker. White arrows and arrowheads indicate detectable and undetectable junctional staining respectively. Scale bar = 10 μm.

(B) IB analysis of fragments of CAMSAP3 (C3) tagged with GFP as preys (ΔCH, ΔCCs, ΔCKK, scheme in Figure 6D) in GST pulldowns using GST-CGNL1(D) as a bait. GST alone is used as a negative bait control, while GFP alone is used as a negative prey control. Numbers indicate migration of pre-stained size markers. Baits are shown in Ponceau-stained image below the IB.

(C) Quantification of junctional labeling intensity for CAMSAP3, ratioed to ZO-1, in mCCD CGNL1-KO cells rescued with FL CGNL1 or CGN or chimeras as shown in Fig. 7B-G. One-way ANOVA with post hoc Dun-nett's test (****$p < 0.0001$, ns) (n = 37-51 cell-cell contacts).

(D) Quantification of GFP-CAMSAP3 constructs bound to the CGNL1 bait ratioed to the input as shown in the IB of Fig. 7I. Unpaired t-test (**$p < 0.01$) (n = 3).

Journal of Cell Science • Supplementary information

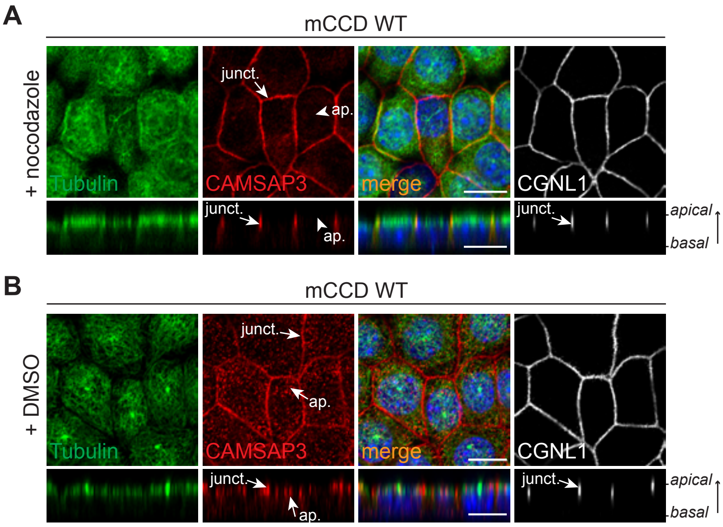

Fig. S7. CAMSAP3 binds to CGNL1 independently of the integrity of the microtubule cytoskeleton.

(A-B) IF microscopy analysis of the localization of CAMSAP3 (red) in mCCD WT cells grown on Transwells and treated with either nocodazole (A) or DMSO (B, negative control). CGNL1 (white) is used as a junctional marker, and tubulin (green) labels microtubules. Z sections were taken at the horizontal middle positions and are shown below XY images. Junctional ("junct.") and apical ("ap.") localizations are indicated. Arrows and arrowheads indicate detectable and decreased/undetectable labeling, respectively. Scale bars = 10 µm.

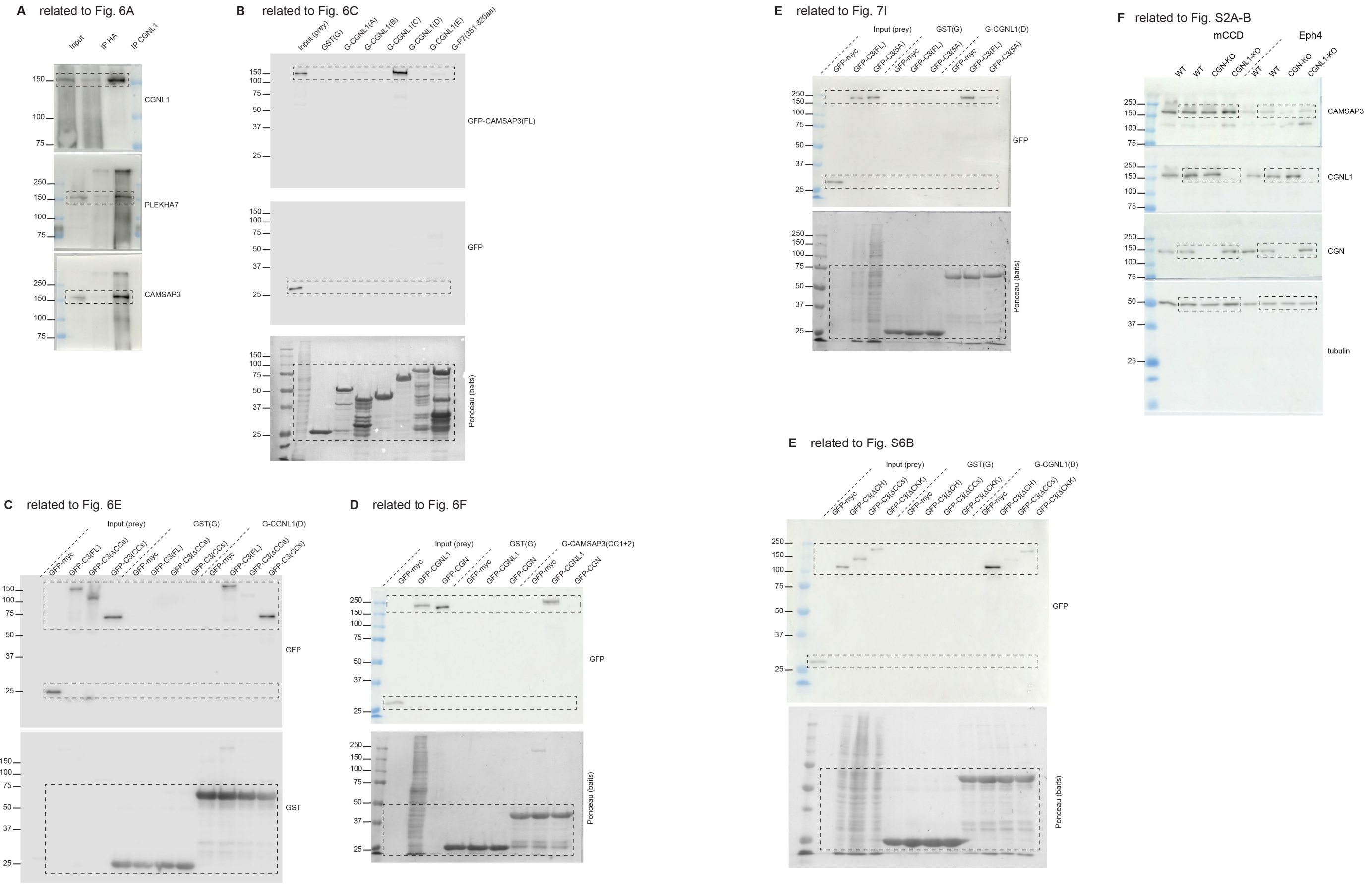

Figure S8: Blot transparency.

Table S1.

REAGENT or RESOURCE	SOURCE	IDENTIFIER
Antibodies		
Mouse monoclonal anti-GFP	Roche	Cat# 11814460001, RRID: AB_390913
Mouse monoclonal anti-HA	Thermo Fisher Scientific	Cat# 32-6700, RRID: AB_2533092
Rabbit polyclonal anti-CAMSAP3	Prof. M. Takeichi, RIKEN University (Meng et al, 2008; Tanaka et al, 2012)	
Guinea pig polyclonal anti-CAMSAP3	Citi Laboratory, This paper	SZC112
Rabbit polyclonal anti-cingulin	Citi Laboratory, (Cardellini et al., 1996)	C532
Mouse monoclonal anti-cingulin	Citi Laboratory	22BD5A1
Rabbit polyclonal anti-paracingulin	Citi Laboratory, (Pulimeno et al., 2011)	20893
Rabbit polyclonal anti-paracingulin	Citi Laboratory, (Guillemot et al., 2008b)	821
Mouse monoclonal anti-paracingulin	Santa Cruz	Cat# sc-377525
Rat monoclonal anti-ZO-1	Prof. D. Goodenough, Harvard Medical School	R40.76, RRID: AB_2205518
Guinea pig polyclonal anti-PLEKHA7	Citi Laboratory, (Guerrera et al., 2016)	GP2737
Rabbit polyclonal anti-PLEKHA7	Citi Laboratory, (Pulimeno et al., 2010)	R30388
Mouse monoclonal anti-E-cadherin	BD Biosciences	Cat# BD 610181, RRID: AB_397580
Rat monoclonal anti-E-cadherin	Thermo Fisher Scientific	Cat# 13-1900, RRID: AB_2533005
Mouse monoclonal anti-β-tubulin	Thermo Fisher Scientific	Cat# 32-2600, RRID: AB_2533072

Rabbit polyclonal anti-α-tubulin	Abcam	Cat# ab18251, RRID: AB_2210057
Guinea pig polyclonal anti-α-tubulin	Geneva Antibody Facility, (Guerreiro and Meraldi, 2019)	scFv-F2C ABCD_AA345
Guinea pig polyclonal anti-β-tubulin	Geneva Antibody Facility, (Guerreiro and Meraldi, 2019)	scFv-S11B ABCD_AA344
Rabbit polyclonal anti-laminin	Sigma-Aldrich	Cat# L9393, RRID: AB_477163
Rabbit polyclonal anti-MACF1	Abcam	Cat# ab117418, RRID: AB_10898474
Rabbit polyclonal anti-polyglutamate chain (polyE)	AdipoGen	Cat# AG-25B-0030, RRID: AB_2490540
Alexa Fluor 488-AffiniPure Donkey Anti-Rabbit IgG	Jackson Laboratory	Cat# 711-545-152, RRID: AB_2313584
Alexa Fluor 488-AffiniPure Donkey Anti-Mouse IgG	Jackson Laboratory	Cat# 715-546-150, RRID: AB_2340849
Alexa Fluor 488-AffiniPure Donkey Anti-Guinea Pig IgG	Jackson Laboratory	Cat# 706-546-148, RRID: AB_2340473
Alexa Fluor 488-AffiniPure Donkey Anti-rat IgG	Jackson Laboratory	Cat# 712-546-150, RRID: AB_2340685
Cy3-AffiniPure Donkey Anti-Rabbit IgG	Jackson Laboratory	Cat# 711-165-152, RRID: AB_2307443
Cy3-AffiniPure Donkey Anti-Mouse IgG	Jackson Laboratory	Cat# 715-165-151, RRID: AB_2315777
Cy3-AffiniPure Donkey Anti-Rat IgG	Jackson Laboratory	Cat# 712-166-150, RRID: AB_2340668
Alexa Fluor 647-AffiniPure Donkey Anti-Guinea Pig IgG	Jackson Laboratory	Cat# 706-605-148, RRID: AB_2340476

Journal of Cell Science • Supplementary information

Alexa Fluor 647-AffiniPure Donkey Anti-Rabbit IgG	Jackson Laboratory	Cat# 711-605-152, RRID: AB_2492288
Cy5-AffiniPure Donkey Anti-Mouse IgG	Jackson Laboratory	Cat# 715-175-150, RRID: AB_2340819
Cy5-AffiniPure Donkey Anti-Rat IgG	Jackson Laboratory	Cat# 712-175-153, RRID: AB_2340672
Anti-Mouse IgG (H+L), HRP Conjugate	Promega	Cat# W4021, RRID: AB_430834
Anti-Rabbit IgG (H+L), HRP Conjugate	Promega	Cat# W4011, RRID: AB_430833
Plasmids		
**CGNL1**		
pGEX4T1-hCGNL1("A", 1-250)	Citi laboratory, (Guillemot et al., 2008b)	S1023
pGEX4T1-hCGNL1("B", 250-420)	Citi laboratory, (Guillemot et al., 2008b)	S1262
pGEX4T1-hCGNL1("C", 421-603)	Citi laboratory, (Guillemot et al., 2008b)	S1020
pGEX4T1-hCGNL1("D", 591-882)	Citi laboratory, (Guillemot et al., 2008b)	S821
pGEX4T1-hCGNL1("E", 884-1302)	Citi laboratory, (Guillemot et al., 2008b)	S1260
pCDNA3.1(-)-cCGNL1(FL)-HA	Citi laboratory, This paper	S2432
pCDNA3.1(-)-GFP-mCGNL1(FL)	Citi laboratory, This paper	S2799
pCDNA3.1(-)-GFP-mCGNL1(mCGN(rod1))	Citi laboratory, This paper	S2800

Journal of Cell Science • Supplementary information

pCDNA3.1(-)-GFP-mCGNL1(Δrod2+tail, 1-880)	Citi laboratory, This paper	S2815
pCDNA3.1(-)-GFP-mCGNL1(FL)	Citi laboratory, This paper	S2875
pCDNA3.1(-)-GFP-mCGNL1(mCGN(rod1))	Citi laboratory, This paper	S2876
**CGN**		
pCDNA3.1(-)-GFP-mCGN(FL)	Citi laboratory, This paper	S2801
pCDNA3.1(-)-GFP-mCGN(mCGNL1(rod1))	Citi laboratory, This paper	S2802
**CAMSAP3**		
pCDNA3.1(-)-GFP-mCAMSAP3(FL)	Citi laboratory, This paper	S2554
pCDNA3.1(-)-GFP-mCAMSAP3(ΔCH, 594-1252)	Citi laboratory, This paper	S2558
pCDNA3.1(-)-GFP-mCAMSAP3(ΔCCs, 1-593 + 948-1252)	Citi laboratory, This paper	S2670
pCDNA3.1(-)-GFP-mCAMSAP3(ΔCKK, 1-1111)	Citi laboratory, This paper	S2671
pCDNA3.1(-)-GFP-mCAMSAP3(CCs, 594-947)	Citi laboratory, This paper	S2672
pGEX4T1-mCAMSAP3(CC1+2, 600-729)	Citi laboratory, This paper	S2796
pGEX4T1-mCAMSAP3(5A)	Citi laboratory, This paper	S2797
**Other constructs**		
pCDNA3.1(-)-GFP-mZO-1(FL)	Citi laboratory, (Rouaud et al 2023)	S2474
pGEX4T1-hPLEKHA7(351-820)	Citi laboratory, This paper	S1193
**Control**		
pCDNA3.1(-)-GFP-myc-his	Citi laboratory, (Guerrera et al., 2016)	S1166
pCDNA3.1(+)-CFP-HA	Citi laboratory, (Spadaro et al., 2014)	S1150
pTRE2hyg-GFP-myc	Citi laboratory, (Paschoud et al., 2014)	S1210

Journal of Cell Science • Supplementary information

Chemicals		
Hygromycin B Gold	InvivoGen	Cat# ant-hg-2
**Critical Commercial Assays and Consumables**		
jetOPTIMUS®	Polyplus	Cat# 117-15
Nocodazole	Sigma-Aldrich	Cat# SML1665
Q5 High fidelity Polymerase	NEB	Cat# M0491L
Proximity Ligation Assay kit	Sigma-Aldrich	Cat# DU092101
Matrigel	BD Biosciences	Cat# 354230
Glass coverslips, 12 mm diameter	Thermo Fisher Scientific/Menzel	Cat# CB00120RAC20MNZ0/CBAD00120RAC20MNZ#0
Glass coverslips, 24 mm diameter, 1.5H	Marienfeld	Cat# 0117640
24 mm Transwell with 0.4 μm pore, either polyester or polycarbonate membrane insert	Corning	Cat# 3450, Cat# 3401
12-well tissue culture plates	Corning	Cat# 353043
24-well tissue culture plates	Corning	Cat# 351147
96-well tissue culture plates	Greiner Bio-One	Cat# 655 180
35-mm Nunc™ Glass Bottom Dishes	Thermo Fisher Scientific	Cat# 150682
Pierce Protease inhibitor cocktail	Thermo Fisher Scientific	A32963
Pierce glutathione magnetic agarose beads	Thermo Fisher Scientific	Cat# 78602
Fluoromount-G	SouthernBiotech	Cat# 0100-01
ProLong Gold	Thermo Fisher Scientific	Cat# P10144
**Experimental Models: Cell Lines**		
Mouse mammary epithelial cell line Eph4 WT	Reichmann Laboratory, University of Zurich, (Fialka et al., 1996)	http://web.expasy.org/cellosaurus/CVCL_0073
Mouse mammary epithelial cell line Eph4 ZO-1-KO	Tsukita Laboratory, Osaka University, (Umeda et al., 2004)	N/A
Mouse mammary epithelial cell line Eph4 CGN-KO	Citi laboratory, (Vasileva et al., 2022)	N/A

Journal of Cell Science • Supplementary information

Mouse mammary epithelial cell line Eph4 CGNL1-KO	Citi laboratory, (Vasileva et al., 2022)	N/A
Mouse Cortical Collecting Duct Cell Line (mCCD) WT N64-Tet-ON	Feraille Laboratory, Unige, (Wang et al., 2014)	N/A
Mouse Cortical Collecting Duct Cell Line CGN-KO	Citi laboratory, (Vasileva et al., 2022)	N/A
Mouse Cortical Collecting Duct Cell Line CGNL1-KO	Citi laboratory, (Vasileva et al., 2022)	N/A
Mouse Cortical Collecting Duct Cell Line PLEKHA7-KO	Citi laboratory, (Shah et al. 2018)	N/A
Experimental Models: Organisms/Strains		
BL21 Competent cells	NEB	Cat# C2530H
DH5-alpha Competent cells	Thermo Fisher Scientific	Cat# 18265017
DH10B Competent cells	Thermo Fisher Scientific	Cat# 18297010
Peptides		
mCAMSAP3 peptide sequence for antibody production: SRLPGSRERDWENG (aa 1085-1098)	This paper (Eurogentec)	N/A
Software and Algorithms		
Image J/FIJI	NIH	imagej.nih.gov/ij/
Adobe Photoshop	Adobe	RRID: SCR _014199
Adobe Illustrator	Adobe	RRID: SCR _010279
Prism 8	GraphPad	RRID: SCR _002798
SnapGene	N/A	RRID: SCR _015052

Journal of Cell Science • Supplementary information

Table S2. CGNL1 and PLEKHA-KO mice are born at Mendelian ratios.

	**Offspring**		
**Parental cross**	Genotype	Number of mice	Percentage (%)
CGNL1 +/- x CGNL1 +/-	-/-	190	24.1
	+/+	198	25.1
	+/-	402	50.9
	**total**	**790**	**100.0**
PLEKHA7 +/- x PLEKHA7 +/-	-/-	30	21.9
	+/+	37	27.0
	+/-	70	51.1
	**total**	**137**	**100.0**

References

Cardellini, P., Davanzo, G. and Citi, S. (1996). Tight junctions in early amphibian development: detection of junctional cingulin from the 2-cell stage and its localization at the boundary of distinct membrane domains in dividing blastomeres in low calcium. *Dev. Dyn.* **207**, 104-113. doi:10.1002/(SICI)1097-0177(199609)207:1<104::AID-AJA10>3.0.CO;2-0

Fialka, I., Schwarz, H., Reichmann, E., Oft, M., Busslinger, M. and Beug, H. (1996). The estrogen-dependent c-JunER protein causes a reversible loss of mammary epithelial cell polarity involving a destabilization of adherens junctions. *J. Cell Biol.* **132**, 1115-1132. doi:10.1083/jcb.132.6.1115

Guerreiro, A. and Meraldi, P. (2019). AA344 and AA345 antibodies recognize the microtubule network in human cells by immunofluorescence. *Antibody Rep.* **2**, e17. doi:10.24450/journals/abrep.2019.e17

Guerrera, D., Shah, J., Vasileva, E., Sluysmans, S., Méan, I., Jond, L., Poser, I., Mann, M., Hyman, A. A. and Citi, S. (2016). PLEKHA7 recruits PDZD11 to adherens junctions to stabilize nectins. *J. Biol. Chem.* **291**, 11016-11029. doi:10.1074/jbc.M115.712935

Paschoud, S., Jond, L., Guerrera, D. and Citi, S. (2014). PLEKHA7 modulates epithelial tight junction barrier function. *Tissue Barriers* **2**, e28755. doi:10.4161/tisb.28755

Spadaro, D., Tapia, R., Jond, L., Sudol, M., Fanning, A. S. and Citi, S. (2014). ZO proteins redundantly regulate the transcription factor DbpA/ZONAB. *J. Biol. Chem.* **289**, 22500-22511. doi:10.1074/jbc.M114.556449

Umeda, K., Matsui, T., Nakayama, M., Furuse, K., Sasaki, H., Furuse, M. and Tsukita, S. (2004). Establishment and characterization of cultured epithelial cellslacking expression of ZO-1. *J. Biol. Chem.* **279**, 44785-44794. doi:10.1074/jbc.M406563200

Wang, Y.-B., Leroy, V., Maunsbach, A. B., Doucet, A., Hasler, U., Dizin, E.,Ernandez, T., de Seigneux, S., Martin, P.-Y. and Feraille, E. (2014). Sodium transport is modulated by p38 kinase-dependent cross-talk between ENaC and Na,K-ATPase in collecting duct principal cells. *J. Am. Soc. Nephrol.* **25**, 250-259. doi:10.1681/ASN.2013040429

Journal of Cell Science • Supplementary information
