# Supplementary material for: Paracingulin recruits CAMSAP3 to tight junctions and regulates microtubule and polarized epithelial cell organization
Source: J Cell Sci. 2023 May 15;137(5):jcs260745. doi: 10.1242/jcs.260745 (PMC10184829; doi:10.1242/jcs.260745)
Supplement: Supplementary file 1 [file joces-137-260745_review_history.pdf]

Journal of Cell Science

Paracingulin recruits CAMSAP3 to tight junctions and regulates microtubule and polarized epithelial organization

Arielle Flinois, Isabelle Méan, Annick Mutero-Maeda, Laurent Guillemot and Sandra Citi
DOI: 10.1242/jcs.260745

Editor: David Bryant

Review timeline
Original submission: 25 October 2022
Editorial decision: 5 December 2022
First revision received: 22 February 2023
Editorial decision: 13 March 2023
Second revision received: 16 March 2023
Accepted: 17 March 2023

Original submission

First decision letter

MS ID#: JOCES/2022/260745

MS TITLE: Paracingulin recruits CAMSAP3 to tight junctions and regulates microtubule organization and polarized epithelial architecture

AUTHORS: Arielle Flinois, Isabelle Mean, Annick Mutero-Maeda, Laurent Guillemot, and Sandra Citi
ARTICLE TYPE: Research Article

We have now reached a decision on the above manuscript, based on the input of three expert reviewers.

To see the reviewers' reports and a copy of this decision letter, please go to: https://submit-jcs.biologists.org and click on the 'Manuscripts with Decisions' queue in the Author Area. (Corresponding author only has access to reviews.)

As you will see, the reviewers all express enthusiasm for your work, but raise criticisms that prevent me from accepting the paper at this stage. They suggest, however, that a revised version might prove acceptable, if you can address their concerns. If you think that you can deal satisfactorily with the criticisms on revision, I would be pleased to see a revised manuscript. We would then return it to the reviewers.

Please ensure that you clearly highlight all changes made in the revised manuscript. Please avoid using 'Tracked changes' in Word files as these are lost in PDF conversion.

I should be grateful if you would also provide a point-by-point response detailing how you have dealt with the points raised by the reviewers in the 'Response to Reviewers' box. Please attend to all of the reviewers' comments. If you do not agree with any of their criticisms or suggestions please explain clearly why this is so.

Reviewer 1

Advance summary and potential significance to field

© 2023. Published by The Company of Biologists under the terms of the Creative Commons Attribution License (https://creativecommons.org/licenses/by/4.0/).

Overall, it is a nice manuscript that focuses on understanding the role of CGNL1 and CAMSAP3 in orchestrating microtubule organization in polarized epithelial cells. Some data is somewhat conformational since some of the findings (CGNL1 interaction with CAMSAP3) have been reported before. Still, it is important study since it systematically analyses the differential roles of CGNL1 and PLEKHA7 in recruiting CAMSAP3 and organizing microtubules. Most data is very strong and use of couple different cell lines, as well as in vivo model, makes it quite convincing. The manuscript does have few issues (see listed below), mostly associated with quantifications and statistical analyses. All of them should be easy to address.

1) Figure 1-2. Overall, very nice and very convincing data. However, it is unclear to me why CAMSAP3 is completely cytosolic in ZO1-KO cells? In those cells CGLN1 is still recruited to AJs via binding to PLEKHA7. Thus, I would expect CAMSAP3 (if it is recruited via binding to CGLN1) to go to AJ.

2) Figure 3A-C. In these images CAMSAP3 and ZO1 has very limited co-localization (in both wt and CGNL1-KO cells). Quantification should be provided about this co-localization.

3) Figure 4A-B. Defects are not super convincing. Higher magnification images would be useful (they can clearly do it using in vivo tissues as shown in Figure 5). Data also needs quantification.

4) Figure 6F-K. Quantification is needed before one can interpret the data.

5) Figure 7F. GFP-CAMSAP3-5A mutants do not seem to localize with MTs anymore. That rises the concern that this mutation affected overall CAMSAP3 structure, making it hard to interpret. At the very least, authors need to show that this CAMSAP3 mutant can bind some other known CAMSAP3 interactors.

6) It is important that in the quantifications (where individual cells are plotted as separate data points, for example see Figure 2C or F) all data is color coded so reviewer can determine how different is the data from different experimental repeats. That is especially important since it appears (based on Statistical analysis section in Methods) that some experiments were only repeated twice.

Comments for the author

see above

Reviewer 2

Advance summary and potential significance to field

This study addresses the mechanisms of microtubule minus-end organization at the cell-cell junctions in epithelial cells.
Previous well-cited work has proposed a mechanism whereby a scaffolding protein PLEKHA7 recruited microtubule minus ends decorated by CAMSAP3 to adherens junctions to organise apico-basal microtubule arrays in a cell culture model of intestinal cells, Caco2. Later work showed that the main mechanism actually responsible for the generation of apico-basal microtubule polarity in intestinal epithelium depends on CAMSAP3 binding to the apical cortex through spectraplakin MACF1/ACF7. In the current study, the authors re-examine microtubule minus-end organization at cell-cell junctions in several epithelial cell models and in knockout mice. The authors show that a protein bound to tight junctions, Paracingulin (CGNL1), is responsible for junctional recruitment of CAMSAP3 and microtubule organization. The great strength of this study is the use of multiple cell and mouse knockout models, as well as biochemical assays characterizing CAMSAP3-CGNL1 interaction in detail. Most of the experiments are very well controlled, the overall quality of the images is good and appropriate quantitative analysis is included. Based on these strengths, the study represents an excellent addition to the field, and is certainly suitable for publication in J Cell Sci.

© 2023. Published by The Company of Biologists under the terms of the Creative Commons Attribution License (https://creativecommons.org/licenses/by/4.0/).

Comments for the author

There are several points that would require clarifications and additional controls, to ensure that the conclusions are fully supported by data.

1. The major functional readout used throughout the paper is the localization of CAMSAP3, and it would be important to establish the specificity of the antibodies used, by using either a knockout or depletion of CAMSAP3. There are several types of localization detected – at the junctions, in the cytoplasm and in the nucleus (e.g., Fig. 2B), and it would be important to determine how specific they are. Even more importantly, it would be good to know whether CAMSAP3 is continuously distributed along CGNL1-positive junctions or whether it is specifically accumulated at the minus-ends of microtubules colocalizing with the junctions. The images in Figure 3 imply the latter (but their quality is a bit limited, as there are numerous CAMSAP3 dots which are not colocalizing either with the junctions or microtubules particularly in WT cells), whereas all the other images in the paper (including rescue experiments in Figure 7) support the former idea. Two not-too-complicated ways of addressing this question further are through microtubule disassembly by nocodazole, to check if CAMSAP3 would still localize to junctions when microtubules are absent, and by more detailed imaging of GFP-CAMSAP3 in the rescue experiments.

2. It is interesting that in cells lacking CGNL1, microtubules at the junctions become more abundant. This point is hardly discussed although it represents an unexpected outcome. It would be important that the authors provide a clearer view of the overall organization of microtubules in WT and CGNL1-knockout cells. A scheme illustrating such organization at the end of the paper would seem appropriate. Do the authors think that binding of CAMSAP3-decorated microtubule minus ends to the junctions promotes their organization perpendicular to the junctions? Some additional discussion of this point would be welcome.

3. A great strength of this study is the generation of two new mouse knockout models. Understandably, the authors would like to save detailed phenotypic characterization of these knockout mice for another study. Still, some short description of the viability and major phenotypes of the mice related to the analyzed tissues would seem appropriate.

Reviewer 3

Advance summary and potential significance to field

The manuscript by Flinois et al., explores the role of junctional proteins in epithelial microtubule organisation and cell morphology in-vitro and in-vivo.
They define the molecular interactions between CGNL1 and the microtubule minus end protein CAMSAP3 at tight junctions, independent of the AJ protein PLEKHA7.
The strength of the study is the classic combination of in-vivo and in-vitro models, with quantitative imaging approaches, and super resolution microscopy complemented by biochemical analyses. It describes a previously unappreciated relationship between the TJ protein CGNL1 and the regulation of the microtubule cytoskeleton organisation in epithelial cells. However, it could be strengthened by including stronger critique of the effects on cell shape/architecture in the absence of CGNL1.

Comments for the author

I have very few experimental recommendations as the data was clear and quantitated sufficiently to support the statements. However, in places, the author?s statements are not supported by the data and I would recommend rewording.

Microtubule organisation in response to CGNL1-KO In figure 3 where authors are imaging the apical microtubule network in Eph4 cells. They state "peri-junctional tubulin labelling was more intense in CGNL1-KO cells suggesting a collapse of MTs into peri-junctional bundles".
The data as it stands doesn't support this statement. I'd recommend softening it to "suggesting an alteration in the apical organisation of the microtubule network".

© 2023. Published by The Company of Biologists under the terms of the Creative Commons Attribution License (https://creativecommons.org/licenses/by/4.0/).

Also "controls the organization of MTs" soften to "contributes"

Loss of cellular and tissue architecture The authors state that there is a loss of tissue architecture, but don't ever clarify what that is. Yes, proteins change localisation- but what does this mean for the cell or tissue shape or function?

For example: In the CCGNL1 KO cells, if the apical microtubule organisation is lost ("collapsed") and the microtubules are "spatially separated" from TJ (figure 3A), what was the consequence on the cellular architecture? Was there a loss of cell height? Change in cell size? nuclear orientation/shape?

In Figure 4- yes there is a loss of nuclear apex distance- but what does that mean at the cellular/tissue scale?

Toya et al., PNAS figure 3 use Caco2 cysts and show a change in architecture (lumen number/size, acini size) in CAMSAP3 siRNA acini. Do the cell lines featured in this manuscript grow as polarised 3D cysts? If so- could a 3D acini model be used to investigate changes in architecture?

I might have misunderstood 3H. ZO1 intensity should be quantitated at the junction for both WT and CGNL1-KO. A simple line scan of existing images.
Particularly as previous reports show ZO3 increases in CGNL1-depleted cells
https://doi.org/10.1091/mbc.e08-06-0558

The manuscript clarity.
The manuscript was dense in sections, and difficult to understand as several parallel concepts were in one sentence. There was *way* too many acronyms, I would recommend at least spelling out microtubule.

Colour-blind friendly images
I would recommend changing red to magenta
Figure 3A-B red arrows in magnified inset
-make these yellow or another colour- not red/the same as the IF pseudo colour.
It was difficult to see.

Figure 3 A-C
keep the position of CAMSAP in the panel consistent. i.e. it is on the bottom on B and C- do the same for A

Typographical errors
Line 67 zonula adhaerens, should read zonula adheren
Latin- either italics or hyphen de novo should read de-novo or de novo
"in mice" should read "in tissue"

First revision

<u>Author response to reviewers' comments</u>

Response to reviewers

Reviewer 1
Advance Summary and Potential Significance to Field:
Overall, it is a nice manuscript that focuses on understanding the role of CGNL1 and CAMSAP3 in orchestrating microtubule organization in polarized epithelial cells. Some data is somewhat conformational, since some of the findings (CGNL1 interaction with CAMSAP3) have been reported before. Still, it is important study since it systematically analyses the differential roles of CGNL1 and PLEKHA7 in recruiting CAMSAP3 and organizing microtubules. Most data is very strong and use of couple different cell lines, as well as in vivo model, makes it quite convincing. The manuscript

© 2023. Published by The Company of Biologists under the terms of the Creative Commons Attribution License (https://creativecommons.org/licenses/by/4.0/).

does have few issues (see listed below), mostly associated with quantifications and statistical analyses. All of them should be easy to address.

1) Figure 1-2. Overall, very nice and very convincing data. However, it is unclear to me why CAMSAP3 is completely cytosolic in ZO1-KO cells? In those cells CGLN1 is still recruited to AJs via binding to PLEKHA7. Thus, I would expect CAMSAP3 (if it is recruited via binding to CGLN1) to go to AJ.

Response. *In ZO-1-KO Eph4 cells there is a faint residual junctional staining for CAMSAP3, that could be attributed to the PLEKHA7-associated CGNL1. However, quantifications indicate that the contribution of such AJ-associated pool of CGNL1 to CAMSAP3 recruitment is minor. This could be due to multivalent interactions or post-translational modifications of CGNL1 at AJ. The text of results and discussion was revised to illustrate this point more clearly. On the other hand, in mCCD cells the KO of PLEKHA7 has no effect on junctional CGNL1/CAMSAP3 labeling, suggesting that all CGNL1 is associated with TJ, and only the TJ (ZO-1)-associated pool of CGNL1 recruits CAMSAP3.*

2) Figure 3A-C. In these images CAMSAP3 and ZO1 has very limited co-localization (in both wt and CGNL1-KO cells). Quantification should be provided about this co-localization.

Response. *CAMSAP3 and ZO-1 actually do not co-localize, not only because they do not interact together, but especially because ZO-1 is localized very close to the TJ membrane (approximately 25 nm), whereas the rod of CGNL1 (where CAMSAP3 binds) is located at a much higher distance from the TJ membrane (approximately 200 nm). ZO-1 is a large molecule, the antibody to ZO-1 binds to the alpha domain (close to the membrane), CGNL1 binds to the extreme C-terminus of ZO-1 (ZU5 domain) and CGNL1 has an asymmetric shape. Thus, ZO-1 is being usedhere exclusively as a spatial marker of the peri-junctional region when comparing the localization of CAMSAP3/microtubules in WT and CGNL1-KO cells. Because ZO-1 and CAMSAP3 do not co-localize precisely, no quantification can be made. The only quantification we can do is the number of CAMSAP3-decorated MT in the peri-junctional region, e.g. within approximately 200 nm (=1 micron in expanded images) of the ZO-1 signal.*

3) Figure 4A-B. Defects are not super convincing. Higher magnification images would be useful (they can clearly do it using in vivo tissues as shown in Figure 5). Data also needs quantification.

Response. *High magnification insets were added for Fig. 4A-B. This is a qualitative result about either absence (WT, PLEKHA7-KO) or presence (CGNL1-KO) of detectable cytoplasmic/lateral labeling for CAMSAP3 in sectioned intestinal epithelia (see arrows and arrowheads in magnified insets.*

4) Figure 6F-K. Quantification is needed before one can interpret the data.

Response. *Quantifications were carried out and are shown in new panel C in revised Fig. S6.*

5) Figure 7F. GFP-CAMSAP3-5A mutants do not seem to localize with MTs anymore. That rises the concern that this mutation affected overall CAMSAP3 structure, making it hard to interpret. At the very least, authors need to show that this CAMSAP3 mutant can bind some other known CAMSAP3 interactors.

Response. *Toya et al. (PNAS 2016) already showed that this mutant still interacts with MT, thus its structure is not affected by the mutation in a way to impact on its interaction with MTs. The text of the Discussion was revised to make this clear.*

6) It is important that in the quantifications (where individual cells are plotted as separate data points, for example see Figure 2C or F) all data is color coded so reviewer can determine how different is the data from different experimental repeats. That is especially important since it appears (based on Statistical analysis section in Methods) that some experiments were only repeated twice.

Response. *We thank this Reviewer for this suggestion. A color code was used for different experimental repeats when showing individual data points.*

© 2023. Published by The Company of Biologists under the terms of the Creative Commons Attribution License (https://creativecommons.org/licenses/by/4.0/).

Reviewer 1 Comments for the Author:
see above

Reviewer 2
Advance Summary and Potential Significance to Field:
This study addresses the mechanisms of microtubule minus-end organization at the cell-cell junctions in epithelial cells.
Previous well-cited work has proposed a mechanism whereby a scaffolding protein PLEKHA7 recruited microtubule minus ends decorated by CAMSAP3 to adherens junctions to organise apico-basal microtubule arrays in a cell culture model of intestinal cells, Caco2. Later work showed that the main mechanism actually responsible for the generation of apico- basal microtubule polarity in intestinal epithelium depends on CAMSAP3 binding to the apical cortex through spectraplakin MACF1/ACF7.
In the current study, the authors re-examine microtubule minus-end organization at cell-cell junctions in several epithelial cell models and in knockout mice. The authors show that a protein bound to tight junctions, Paracingulin (CGNL1), is responsible for junctional recruitment of CAMSAP3 and microtubule organization. The great strength of this study is the use of multiple cell and mouse knockout models, as well as biochemical assays characterizing CAMSAP3-CGNL1 interaction in detail. Most of the experiments are very well controlled, the overall quality of the images is good and appropriate quantitative analysis is included. Based on these strengths, the study represents an excellent addition to the field, and is certainly suitable for publication in J Cell Sci.

Reviewer 2 Comments for the Author:
There are several points that would require clarifications and additional controls, to ensure that the conclusions are fully supported by data.

1. The major functional readout used throughout the paper is the localization of CAMSAP3, and it would be important to establish the specificity of the antibodies used, by using either a knockout or depletion of CAMSAP3. There are several types of localization detected – at the junctions, in the cytoplasm and in the nucleus (e.g., Fig. 2B), and it would be important to determine how specific they are.

***Response**. Thank you for this comment. The antibody used for localization of endogenous CAMSAP3 was a kind gift from Prof. Takeichi, and the specificity of this antibody was demonstrated in their publication (Meng et al Cell 2008, Fig. 2E).*

Even more importantly, it would be good to know whether CAMSAP3 is continuously distributed along CGNL1-positive junctions or whether it is specifically accumulated at the minus-ends of microtubules colocalizing with the junctions. The images in Figure 3 imply the latter (but their quality is a bit limited, as there are numerous CAMSAP3 dots which are not colocalizing either with the junctions or microtubules, particularly in WT cells), whereas all the other images in the paper (including rescue experiments in Figure 7) support the former idea. Two not-too-complicated ways of addressing this question further are through microtubule disassembly by nocodazole, to check if CAMSAP3 would still localize to junctions when microtubules are absent, and by more detailed imaging of GFP-CAMSAP3 in the rescue experiments.

***Response**. We thank this Reviewer for this insightful comment. We carried out the analysis of CAMSAP3 localization upon treatment of cells with nocodazole, and the results (new Fig- S7) show that CAMSAP3 still localizes at junctions, whereas the cytoplasmic/apical pool becomes undetectable upon MT depolymerization. This allows us to conclude that CAMSAP3 can bind to CGNL1 independently of MT cytoskeleton integrity, and that the 5A mutation affects the binding to CGNL1 and not to MT. The text was revised accordingly.*

2. It is interesting that in cells lacking CGNL1, microtubules at the junctions become more abundant. This point is hardly discussed, although it represents an unexpected outcome. It would be important that the authors provide a clearer view of the overall organization of microtubules in WT and CGNL1-knockout cells. A scheme illustrating such organization at the end of the paper would seem appropriate. Do the authors think that binding of CAMSAP3-decorated microtubule minus ends to the junctions promotes their organization perpendicular to the junctions? Some

© 2023. Published by The Company of Biologists under the terms of the Creative Commons Attribution License (https://creativecommons.org/licenses/by/4.0/).

additional discussion of this point would be welcome.

Response. *We thank this Reviewer for this suggestion. A scheme of MT and CAMSAP3 organization/localization in WT and CGNL1-KO cells was added (new Fig. 8K), with a summary at the beginning of the Discussion.*

3. A great strength of this study is the generation of two new mouse knockout models. Understandably, the authors would like to save detailed phenotypic characterization of these knockout mice for another study. Still, some short description of the viability and major phenotypes of the mice related to the analyzed tissues would seem appropriate.

Response. *Additional information about the KO mice was included in the methods section. As the Reviewer correctly understands, mouse tissues are used here to validate the phenotypes obtained in cultured cells, and additional phenotypic characterization of the phenotypes of these mice belongs to future studies.*

Reviewer 3

Advance Summary and Potential Significance to Field:
The manuscript by Flinois et al., explores the role of junctional proteins in epithelial microtubule organisation and cell morphology in-vitro and in-vivo. They define the molecular interactions between CGNL1 and the microtubule minus end protein CAMSAP3 at tight junctions, independent of the AJ protein PLEKHA7. The strength of the study is the classic combination of in-vivo and in-vitro models, with quantitative imaging approaches, and super resolution microscopy, complemented by biochemical analyses.
It describes a previously unappreciated relationship between the TJ protein CGNL1 and the regulation of the microtubule cytoskeleton organisation in epithelial cells. However, it could be strengthened by including stronger critique of the effects on cell shape/architecture in the absence of CGNL1.

Reviewer 3 Comments for the Author:

I have very few experimental recommendations as the data was clear and quantitated sufficiently to support the statements. However, in places, the author’s statements are not supported by the data and I would recommend rewording.

Microtubule organisation in response to CGNL1-KO
In figure 3 where authors are imaging the apical microtubule network in Eph4 cells. They state "peri-junctional tubulin labelling was more intense in CGNL1-KO cells suggesting a collapse of MTs into peri-junctional bundles".
The data as it stands doesn't support this statement. I'd recommend softening it to "suggesting an alteration in the apical organisation of the microtubule network".

Also "controls the organization of MTs" soften to "contributes"

Response. *The text was revised as suggested by this Reviewer. In addition, we carried out an important additional rescue experiment, using CGNL1/CGN chimeras, to ask whether the effect of KO of CGNL1 on MT organization was dependent on CAMSAP3 interaction. The results (panels L-P in revised Fig. 6) show that CGNL1 controls MT organization independently of CAMSAP3. Since CGNL1 binds directly to MT (Vasileva et al 2018) this observation allows us to make additional comments (revised Discussion) about different mechanisms through which CGNL1 may control MT organization.*

Loss of cellular and tissue architecture
The authors state that there is a loss of tissue architecture, but don't ever clarify what that is. Yes, proteins change localisation- but what does this mean for the cell or tissue shape or function?

Response. *We observe a consistent change in the apico-basal positioning of the nuclei in intestinal epithelial cells of CGNL1-KO mice. In this sense, it is an alteration of cell architecture. The text*

© 2023. Published by The Company of Biologists under the terms of the Creative Commons Attribution License (https://creativecommons.org/licenses/by/4.0/).

was revised to make this clear. We have not examined the overall shape or function of any tissue yet, this will belong to a future phenotypic characterization of the mice.

For example: In the CCGNL1 KO cells, if the apical microtubule organisation is lost ("collapsed") and the microtubules are "spatially separated" from TJ (figure 3A), what was the consequence on the cellular architecture? Was there a loss of cell height? Change in cell size? nuclear orientation/shape?

Response. *It is difficult to measure cell height in cysts, since not all cysts have the same size. Effects on cellular architecture were also hard to assess on cultured cells, as the polarization is much less pronounced than in columnar epithelial tissues. Cell size differences were not detected, either, and the nucleus occupies the entirety of the cell, making it impossible to find any difference between WT and KO cells. Intestinal epithelial (columnar) cells are ideally suited for these measures, this is why we generated and analyzed KO mice.*

In Figure 4- yes there is a loss of nuclear apex distance- but what does that mean at the cellular/tissue scale?

Response. *At the cellular scale, this means that the overall cell architecture is perturbed by the disorganization of cytoplasmic microtubules, as described in Toya et al (PNAS 2016) for CAMSAP3 mutant mice. The implication of a similar cellular phenotype (for CAMSAP3 mutant mice) at the tissue level were described in Mitsuhata et al (Sci. Rep. 2021), for example cysts in kidney proximal convoluted tubules. As stated above, a detailed phenotypic analysis of CGNL1-KO epithelial tissues/organs has not been carried out yet and will be the object of future studies. We did not notice altered histology of the kidney of CGNL1-KO micey, but we will expand our analyses in future work. The present study is focused on cells and subcellular structures, not on tissues and organs.*

Toya et al., PNAS figure 3 use Caco2 cysts and show a change in architecture (lumen number/size, acini size) in CAMSAP3 siRNA acini. Do the cell lines featured in this manuscript grow as polarised 3D cysts? If so- could a 3D acini model be used to investigate changes in architecture?

Response. *We thank this Reviewer for this constructive comment. We carried out analysis of WT and CGNL1-KO cysts grown in Matrigel (new panels in Figure 1E-I and Fig. 8D-J). The KO of CGNL1 has an impact on cyst morphogenesis, and this phenotype requires the Rod-1 region of CGNL1 for a rescue.*

I might have misunderstood 3H. ZO1 intensity should be quantitated at the junction for both WT and CGNL1-KO. A simple line scan of existing images. Particularly as previous reports show ZO3 increases in CGNL1-depleted cells https://doi.org/10.1091/mbc.e08-06-0558

Response. *In this Figure, ZO-1 labeling is exclusively used to identify the outermost cell area. The intensity of ZO-1 and ZO-3 labeling is not affected by KO of CGNL1 in different epithelial cell lines, as shown in our recent paper Vasileva et al. (JBC 2022). Instead, the KO of CGN does reduce both. The publication this Reviewer refers to studies the effect of acute depletion of CGNL1 by shRNA (not KO) and we observed a small increase in ZO-3 mRNA and protein levels, but no change in the intensity of immunofluorescent ZO-3 labeling at junctions.*

The manuscript clarity.
The manuscript was dense in sections, and difficult to understand as several parallel concepts were in one sentence. There was *way* too many acronyms, I would recommend at least spelling out microtubule.

Response. We used microtubule throughout, instead of MT, and reduced acronyms as much as possible.

Colour-blind friendly images
I would recommend changing red to magenta

Response. *Magenta color is very similar to DAPI blue, and we feel the clarity of the images would be affected by changing the color.*

© 2023. Published by The Company of Biologists under the terms of the Creative Commons Attribution License (https://creativecommons.org/licenses/by/4.0/).

Figure 3A-B red arrows in magnified inset-make these yellow or another colour- not red/the same as the IF pseudo colour. It was difficult to see.

Response. *Green and red arrows were modified to have an outer white line in order to make them more visible.*

Figure 3 A-C
keep the position of CAMSAP in the panel consistent. i.e. it is on the bottom on B and C- do the same for A

Response. *The labeling was made consistent for all panels.*

Typographical errors
Line 67 zonula adhaerens, should read zonula adherens
Latin- either italics or hyphen de novo should read de-novo or de novo "in mice" should read "in tissue"

Response. *Thanks. The typos were corrected (except for zonula adhaerens, which is the correct latin spelling- https://www.wordsense.eu/adhaerens/).*

Second decision letter

MS ID#: JOCES/2022/260745

MS TITLE: Paracingulin recruits CAMSAP3 to tight junctions and regulates microtubule and polarized epithelial organization

AUTHORS: Arielle Flinois, Isabelle Mean, Annick Mutero-Maeda, Laurent Guillemot, and Sandra Citi
ARTICLE TYPE: Research Article

We have now reached a decision on the above manuscript.

To see the reviewers' reports and a copy of this decision letter, please go to: https://submit-jcs.biologists.organd click on the 'Manuscripts with Decisions' queue in the Author Area. (Corresponding author only has access to reviews.)

As you will see, all reviewers are extremely favourable regarding the publication of your paper. However, Reviewer 2 maintains a critical point of validation of the anti-CAMSAP3 antibodies, which was raised in their original review. I hope that you will be able to carry this out because I would very much like to be able to accept your paper, depending on this validation.

Please ensure that you clearly highlight all changes made in the revised manuscript. Please avoid using 'Tracked changes' in Word files as these are lost in PDF conversion.

I should be grateful if you would also provide a point-by-point response detailing how you have dealt with the points raised by the reviewers in the 'Response to Reviewers' box. Please attend to all of the reviewers' comments. If you do not agree with any of their criticisms or suggestions please explain clearly why this is so.

Reviewer 1

Advance summary and potential significance to field

Authors have addressed all my concerns and incorporated all suggestions. As the result, in my opinion, this manuscript is now ready for publication.

© 2023. Published by The Company of Biologists under the terms of the Creative Commons Attribution License (https://creativecommons.org/licenses/by/4.0/).

Comments for the author

Authors have addressed all my concerns and incorporated all suggestions. As the result, in my opinion, this manuscript is now ready for publication.

Reviewer 2

Advance summary and potential significance to field

As indicated in my previous review, overall, I find this a strong paper potentially suitable for publication in the Journal of Cell Science.

Comments for the author

Unfortunately, the authors chose to ignore my request to characterize the specificity of the anti-CAMSAP3 antibodies, on which the majority of the conclusions of this paper rests. This is in my view inappropriate because the characterization of these antibodies is described in the Meng et al Cell 2008 paper - the very paper the conclusions of which the authors of the current manuscript bring into question. The specificity of this anti-CAMSAP3 antibody is questionable because nuclear staining is observed (e.g. Fig. 2B), whereas GFP-CAMSAP3 is never found in the cell nucleus. This is an essential point that needs proper clarification before the paper is published. Performing stainings with this antibody in CAMSAP3-depleted or CAMSAP3-knockout epithelial cells that the authors study would address this comment.

Reviewer 3

Advance summary and potential significance to field

The manuscript by Flinois et al., explores the role of junctional proteins in epithelial microtubule organisation and cell morphology in-vitro and in-vivo.
They define the molecular interactions between CGNL1 and the microtubule minus end protein CAMSAP3 at tight junctions, independent of the AJ protein PLEKHA7. The study brings new knowledge regarding the relationship between the microtubule cytoskeleton, cell adhesion and cell morphology/architecture.

Comments for the author

I am happy with how the authors have addressed my concerns.

Second revision

Author response to reviewers' comments

Response to Reviewer2

Reviewer 2 Comments for the Author:
Unfortunately, the authors chose to ignore my request to characterize the specificity of the anti-CAMSAP3 antibodies, on which the majority of the conclusions of this paper rests. This is in my view inappropriate because the characterization of these antibodies is described in the Meng et al Cell 2008 paper - the very paper the conclusions of which the authors of the current manuscript bring into question. The specificity of this anti-CAMSAP3 antibody is questionable because nuclear staining is observed (e.g. Fig. 2B), whereas GFP-CAMSAP3 is never found in the cell nucleus. This is an essential point that needs proper clarification before the paper is published. Performing stainings with this antibody in CAMSAP3-depleted or CAMSAP3- knockout epithelial cells that the authors study would address this comment.

© 2023. Published by The Company of Biologists under the terms of the Creative Commons Attribution License (https://creativecommons.org/licenses/by/4.0/).

Response. *We did not ignore the request to characterize the specificity of the anti-CAMSAP3 antibodies. We responded that the antibodies had already been validated.*
Indeed, the Takeichi lab polyclonal antibodies described in Meng et al 2008 (doi:10.1016/j.cell.2008.09.040) and in Tanaka et al 2012 (doi:10.1073/pnas.121801710) were validated by immunoblot and immunofluorescence analysis of cells subjected to siRNA-mediated depletion of CAMSAP3, as shown in the Figures 2E and S2A-B below (relevant images in red circles):

NOTE: We have removed previously published data that had been provided for the referees.

For this reason, we do not think it is necessary that we repeat already published experiments.

Moreover:

- *The Takeichi lab polyclonal antibodies were raised against <u>mouse</u> CAMSAP3 antigens, so they are bound to recognize the mouse antigen, as they do (mCCD, Eph4 cells-our study, and mouse tissues – see Tanaka et al 2012, Toya et al 2016, and our study), in addition to cross-reacting with human CAMSAP3 (Caco2 cells).*
- *The aminoacid sequences of human and mouse CAMSAP3 are highly similar in the region used to generate the antibodies validated in Tanaka et al 2012 (yellow highlight in the sequence alignment below between human and mouse CAMSAP3) and 100% identical in the peptide region used to generate the antibodies validated in Meng et al 2008 (green highlight in the sequence alignment below).*

NOTE: We have removed unpublished data that had been provided for the referees..

- *The Takeichi lab polyclonal antibodies were used in essentially all the experiments reported in our paper, on which the majority of the conclusions of our paper rest, as the Reviewer correctly states. The exception is Fig. S5C, where we used a home-made guinea-pig polyclonal antibody to label kidney sections in order to do double labeling with anti-polyE rabbit antibodies (NB the labeling was identical using the rabbit polyclonal against CAMSAP3). The specificity of the guinea pig polyclonal antibody is shown below, compared to the antibodies from the Takeichi laboratory (Tanaka et al 2012). The Takeichi lab (Tanaka et al) antibody (raised against residues 596-1076) recognizes the full-length and N-terminal fragment (1-892), whereas our guinea pig antibody (raised against the same peptide antigen used for the rabbit anti-CAMSAP3 described in Meng et al 2008, 100% identity between human and mouse, see alignment above) recognizes the full-length and the C-terminal fragment (948-1252) of CAMSAP3.*

NOTE: We have removed unpublished data that had been provided for the referees in confidence.

Regarding the nuclear staining mentioned by the Reviewer (Fig. 2B), in our experience it can happen that (especially) polyclonal antibodies occasionally give non-specific staining of the nuclei and/or the cytoplasm, depending on fixation procedure, length of incubation, washing steps (length of time, number of washes, composition of buffer), blocking step (length of incubation, composition of blocking buffer), high expression of transgenes which are recognized by cross-reacting secondary antibodies, direct non-specific cross-reactivity of secondary antibodies with nuclear antigens, etc etc. Indeed, in Fig. 2B a similar weak background nuclear staining is visible in the channel of the polyclonal anti-PLEKHA7 antibody. In addition, we see a weak nuclear labeling in the CAMSAP3 channel in Fig. S1B (bottom panel), where there is a high expression of CFP-HA (which we believe is the source of the nuclear non-specific staining in the CAMSAP3 channel). In contrast, in Fig. 1, Fig. 2A, Fig. 4, Fig. 7, Fig. 8, Fig. S1A, Fig. S1B (top panel), Fig. S7 there is no nuclear non-specific background staining with anti-CAMSAP3 polyclonal antibodies. All together we have about *30 immunofluorescence microscopy panels (including XY, Z images of cells, and tissue* sections) with no nuclear labeling by the rabbit polyclonal anti-CAMSAP3, versus 3 panels with a weak nuclear labeling. We feel it is reasonable to conclude that the occasional nuclear labeling is due to non-specific cross-reaction that can rarely happen but does not automatically invalidate the specificity of the reagent. And, most importantly, the presence of the nuclear labeling does not impact the conclusion about the presence/absence of the junctional localization of CAMSAP3.

© 2023. Published by The Company of Biologists under the terms of the Creative Commons Attribution License (https://creativecommons.org/licenses/by/4.0/).

Finally, although our conclusions bring into question the conclusions of Meng et al 2008, e.g. CAMSAP3 is recruited to junctions by CGNL1 and not by PLEKHA7, the discrepancy in the conclusions between the studies has nothing to do with the specificity of the anti-CAMSAP3 antibodies. On the contrary, it is precisely because we used antibodies from the Takeichi laboratory and validated by the Takeichi laboratory that we are confident that our results are not due to antibodies with different, doubtful or non-validated specificity. Rather, as we mention in the Discussion section, the discrepancy in conclusions could be due to the different experimental models used (Caco2 vs mCCD and Eph4 cells, where the localization of CAMSAP3 at confluence is different) and depletion approaches (siRNA vs KO).

In summary, we believe that the evidence that the antibodies are specific and that the junctional localization of CAMSAP3 depends on CGNL1 is extremely solid.

REVISIONS:

1. *We revised the text of the Methods section and Reagents Table, to refer to the validation of the published anti-CAMSAP3 polyclonal antibodies.*
2. *We revised the text of the Methods section regarding the guinea-pig polyclonal antibody.*
3. *We revised the legends, to indicate that the nuclear labeling is likely due to non-specific cross-reactivity.*
4. *To reinforce our conclusions, we also revised Fig. 7, by adding panels showing that exogenous CAMSAP3 does not localize to junctions when expressed in CGNL1-KO cells. The text of the Results section was modified as follows* "Importantly, full-length WT CAMSAP3 was not targeted to junctions in CGNL1-KO cells (Fig. 7K), confirming the role of CGNL1 in the junctional recruitment of CAMSAP3, independently of the use of antibodies to label CAMSAP3." *The text of the Discussion was also revised to underline this new evidence.*

Third decision letter

MS ID#: JOCES/2022/260745

MS TITLE: Paracingulin recruits CAMSAP3 to tight junctions and regulates microtubule and polarized epithelial organization

AUTHORS: Arielle Flinois, Isabelle Mean, Annick Mutero-Maeda, Laurent Guillemot, and Sandra Citi
ARTICLE TYPE: Research Article

I am happy to tell you that your manuscript has been accepted for publication in Journal of Cell Science, pending standard ethics checks.

Reviewer 2

Advance summary and potential significance to field

See my previous review

Comments for the author

Although I would have strongly preferred to see a proper validation of this antibody by the authors of the current manuscript, and although it would have been very easy to do, I do not want to insist on it and further delay publication.

© 2023. Published by The Company of Biologists under the terms of the Creative Commons Attribution License (https://creativecommons.org/licenses/by/4.0/).
